# Molecular Indicators of Isometric Exercise Efficacy in Early Rehabilitation of Older Adults After Total Hip Arthroplasty

**DOI:** 10.3390/ijms27031389

**Published:** 2026-01-30

**Authors:** Elena A. Maksimova, Kirill S. Krasnov, Anatoly S. Senotov, Victor I. Shevchenko, Artem M. Ermakov, Elizaveta S. Zhdanova, Vladimir S. Akatov

**Affiliations:** 1 Institute of Theoretical and Experimental Biophysics, Russian Academy of Sciences, 142290 Pushchino, Russia; 2 Hospital of Pushchino Scientific Center, Russian Academy of Sciences, 142290 Pushchino, Russia; v-shevc68@mail.ru

**Keywords:** total hip arthroplasty, early rehabilitation, isometric exercises, pro-inflammatory interleukins, transcriptome, bioinformatics

## Abstract

Developing molecular methods for assessing the effectiveness of physical rehabilitation remains a pressing task. Our pilot study aimed to assess the utility of the transcriptome analysis of muscle biopsies in evaluating the efficacy of the isometric exercises (IEs) added to the standard protocol in the early rehabilitation of older patients during the initial two weeks post total hip arthroplasty (THA). Blood concentrations of total leukocytes, neutrophils, IL6, IL8, IL1β, myoglobin, etc. were measured, and transcriptome analysis of rectus femoris biopsies from the operated leg was performed before and after (1 and 12 days) THA in women aged 73–77 years. The additional IEs in the experimental rehabilitation group led to a significant acceleration in the recovery of IL6, IL8, and IL1β blood levels to the normal ranges compared to the control group, as confirmed by a Fisher’s exact test for this cytokine combination. The results of Gene Set Enrichment Analysis and Differentially Expressed Gene Analyses for the muscle biopsies point to accelerated resolution of inflammation, along with enhanced activation of genes associated with angiogenesis, lymphangiogenesis, vasodilation, and vasoconstriction in the experimental rehabilitation group compared to the control group. Thus, IL6, IL8, and IL1β blood levels can serve in combination as molecular indicators of the efficacy of early rehabilitation after THA, and transcriptome analysis of the rectus femoris biopsies of the operated leg allows for the revelation of the molecular indicators of regenerative processes in muscle tissue near the surgery area after THA.

## 1. Introduction

Currently, total hip arthroplasty (THA) is considered the “gold standard” for treating late-stage osteoarthritis (OA) of the hip joint. As people live longer, the incidence of OA affecting the major joints of the lower extremities also increases. According to a systematic analysis for the Global Burden of Disease Study 2021, 595 million people had OA in 2020, equal to 7.6% of the global population, with an increase of 132.2% in total cases since 1990. According to these assessments, 453.56 million people aged 55 and older were living with OA in 2021, accounting for 30.39% of this population and contributing to 16.05 million disability-adjusted life years (DALYs) [1,2]. Rehabilitation based on physical exercises is a crucial stage in the treatment of patients after THA. Physical exercises are prescribed from the first day after THA [3]. After the surgery, patients experience loss of muscle strength and functional capacity, as well as long-term postoperative complications [4,5,6]. Early rehabilitation in the first two weeks following THA is critical for the entire subsequent rehabilitation process [7]. Isometric exercises (IEs) are an essential component of early rehabilitation after THA because high-impact joint loading and dynamic exercises are contraindicated during this period. This is particularly relevant for older patients due to age-related changes, comorbidities, and a high risk of complications [8,9]. It is known that contractile activity during IEs induces an increase in sarcoplasmic calcium ion concentration in muscle cells, enhanced nitric oxide (NO) production by muscle cells, transient rise in sodium ion concentration, and decrease in potassium ion concentration. These changes collectively influence gene expression, enhance cytokine secretion by muscle cells, promote cell proliferation and differentiation in muscle tissue, and affect osteogenic differentiation in adjacent bone tissues, thereby activating myogenesis and osteogenesis [10,11,12,13,14,15,16]. However, these general concepts do not allow for the understanding of how specific exercise protocols may enhance regenerative processes [17,18,19]. Therefore, developing new rehabilitation methods is based largely on empirical approaches.

Improving early physical rehabilitation programs for THA patients requires new objective methods to evaluate their effectiveness [17,18,19]. This need arises because traditional assessment methods based on standard functional scales and tests (e.g., visual analog pain scale, 10-m walk test, Lequesne and Harris scales, etc.) are not applicable in the early stages (1–2 weeks) after THA due to patients’ weakened postsurgical condition [3,9]. Our pilot study aimed to assess the potential of the transcriptome analysis of muscle biopsies from the operated limb, along with the blood parameters of elderly patients, to determine the effectiveness of IEs in early rehabilitation following THA. For this aim, during the first 12 days after the THA, the patients performed daily rehabilitation exercises following the standard IE protocol in the control group while following a novel rehabilitation regimen incorporating additional IEs in the experimental group. The clinical parameters of the blood, muscle trauma markers, erythrocyte sedimentation rate (ESR), C-reactive protein (CRP), and the concentration of pro-inflammatory interleukins (IL-6, IL-8, and IL-1β) were evaluated 1 day before the THA, as well as 1 day and 12 days after the THA, for each patient, to identify differences between the groups. At these times, rectus femoris biopsies of the operated leg were also performed for each patient for transcriptomic analysis.

## 2. Results

### 2.1. Clinical and Biochemical Blood Analysis Results in Standard and Experimental Rehabilitation Groups

Clinical and biochemical blood analysis results (Tables S1 and S2) focused primarily on molecular and cellular factors related to postoperative inflammation and muscle trauma. No differences between the control and experimental rehabilitation groups were found for other blood parameters.

**Figure 1:**
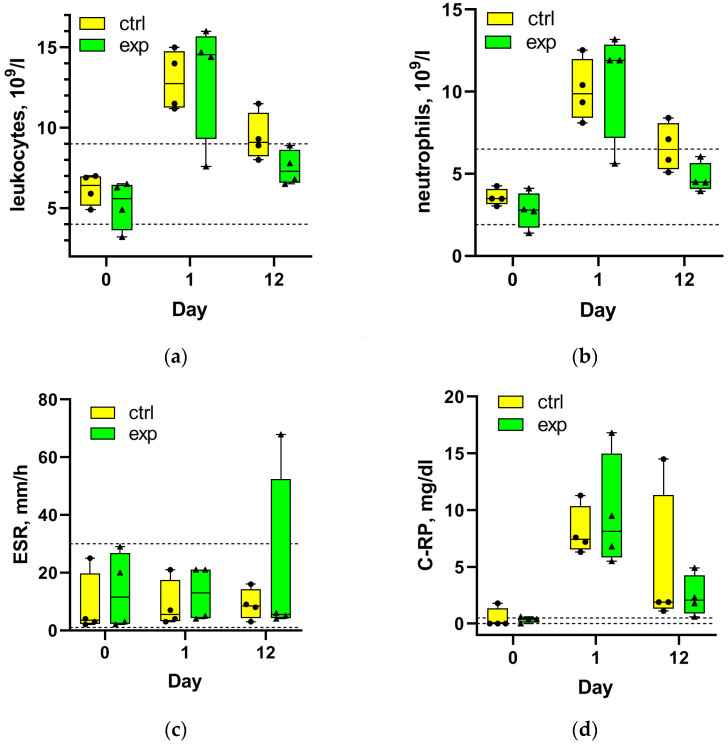
The concentration of total leukocytes (**a**) and neutrophils (**b**), erythrocyte sedimentation rate (ESR) (**c**) and C-reactive protein (CRP) level (**d**) in the blood of patients in the control and experimental rehabilitation groups 1 day before surgery (0) and 1 day (1) and 12 days after total hip arthroplasty (THA). The symbols indicate individual patient parameters. Dotted lines show the upper and lower limits of the normal values for each parameter.

Before surgery (1 day), both total leukocyte (Figure 1a) and neutrophil (Figure 1b) counts were within normal ranges in both groups. One day postsurgery, these values increased significantly (1.5–2 times above normal upper limits). By day 12 post-THA, mean values decreased to near upper normal limits. In the experimental group, all patients had normal values by day 12, while half of the control group remained above normal limits. According to a Mann–Whitney test, no statistically significant differences were found between the control and experimental groups for total leukocyte and neutrophil concentrations either before or 1 and 12 days after THA.

No large changes in ESR were observed at either 1 or 12 days post-THA compared to preoperative values (Figure 1c). A high variability in ESR values was noted in both groups, but all ESR values remained below the upper reference limit except for one patient in the experimental group. No significant differences in ESR between the rehabilitation groups were found (Mann–Whitney test).

Preoperative CRP levels were within normal limits (<0.5 mg/dL) but increased 10–20 times above the upper normal limit 1 day postsurgery (Figure 1d). At day 12, the experimental group showed a mean CRP concentration (2.4 mg/dL) half that of the control group; however, this difference was not significant (Mann–Whitney test), and both values remained well above the upper normal limit.

One day before surgery, muscle trauma markers—myoglobin, aspartate aminotransferase (AST), lactate dehydrogenase (LDH), and creatine phosphokinase (CPK)—were within normal limits (Table S2). One day post-THA, myoglobin and CPK levels increased manyfold and exceeded significantly the upper limits in contrast to AST and LDH. AST levels increased no more than twofold, approaching the upper normal limit, and changes in LDH were even less pronounced. By day 12, concentrations of myoglobin, CPK, and AST returned to preoperative levels in both groups, and LDH was within the normal limits too. No significant differences for trauma markers between the rehabilitation groups were found (Mann–Whitney test).

**Figure 2:**
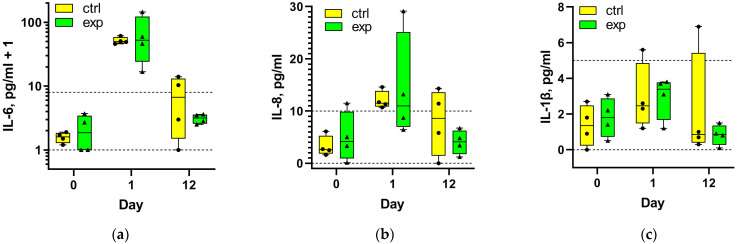
Serum concentrations of interleukins IL-6 (**a**), IL-8 (**b**), and IL-1β (**c**) in patients of the control and experimental rehabilitation groups 1 day before surgery (0) and 1 day (1) and 12 days (12) after THA. Each data point represents an individual patient. Dotted lines indicate the upper and lower limits of the normal reference ranges for each interleukin.

Figure 2 shows the serum concentrations of interleukins IL-6, IL-8, and IL-1β in patients from the control and experimental groups at 1 day presurgery (0) and 1 and 12 days post-THA. Preoperative levels were within normal ranges, except for one experimental patient with slightly elevated IL-8. The most significant increase (up to 100-fold) was observed for IL-6 at day 1 post-THA in both groups (Figure 2a), and all patients exceeded the upper normal limit for IL-6. IL-8 levels also demonstrated a large increase (up to 5-fold) at day 1 post THA, with some patients remaining within normal limits (Figure 2b). IL-1β increased less significantly (mostly within normal limits), except for one patient. By day 12 post-THA, all patients of the experimental group had IL-6, IL-8, and IL-1β levels within normal limits, while in the control group, only half of patients reached normal IL-6 levels, and IL-8 changes were similar. IL-1β concentrations mostly remained at 12-day post-THA within normal limits in the control group, except for one patient. TNFα concentrations remained within normal limits at both 1 day presurgery and 12 days post-THA in both groups. In total, at day 12, four experimental patients had all 12 interleukin measurements within normal limits, whereas in the control group, 7 values were normal and 5 exceeded normal limits. The difference between the groups regarding exceeding normal limits in aggregate was statistically significant (*p* < 0.05) according to a Fisher’s exact test (*p* = 0.037).

### 2.2. Comparative Transcriptomic Analysis of Rectus Femoris Biopsies from the Operated Leg in Patients of the Control and Experimental Rehabilitation Groups

#### 2.2.1. Differentially Expressed Gene (DEG) Analysis Reveals Difference Between Patients of Control and Experimental Rehabilitation Groups

**Figure 3:**
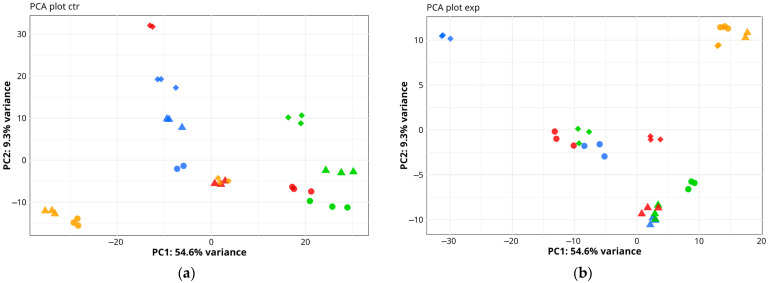
Principal component analysis (PCA) plots of normalized transcriptomic data for muscle biopsy samples taken from patients of the control (**a**) and experimental (**b**) rehabilitation groups at three time points: at the start of the operation (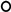) and 1 day (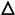) and 12 days (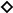) after THA. Data from four patients per group are shown, with individual patients represented by distinct colors. For each muscle sample, up to three sequencing runs were performed, and all resulting data were included in the analysis.

Before analyzing differential gene expression after surgery and rehabilitation, we assessed sample variability. Figure 3 shows the results of principal component analysis (PCA) for patients of the control and experimental rehabilitation groups. The PCA results reveal differences among patient samples at three time points: presurgery and 1 day and 12 days after THA. Principal component 1 (PC1) accounted for 54.6% of gene expression variance, while PC2 accounted for 9.3%. Overall, these findings indicate transcriptomic differences in muscle biopsy samples across all patients—both control and experimental groups—at the three time points (pre-surgery and 1 day and 12 days post-THA).

**Figure 4:**
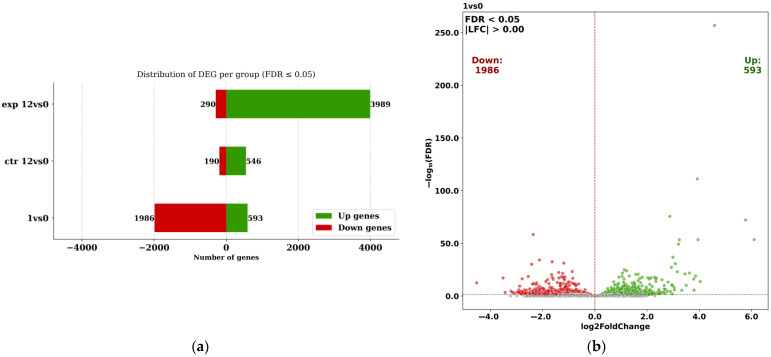

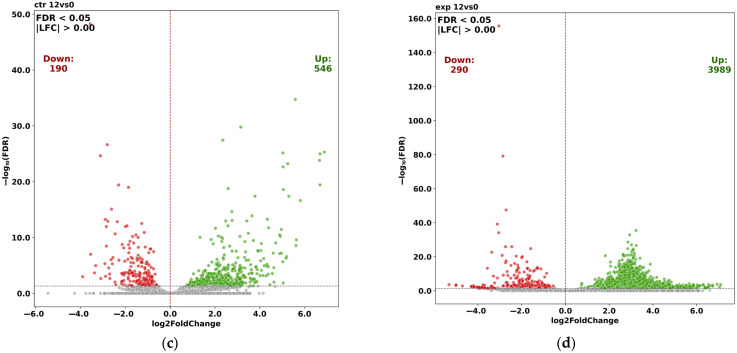
The number of genes with decreased and increased expression compared to preoperative activity 1 day after THA in the control and experimental rehabilitation groups together, designated as set 1vs0, and in the control and experimental rehabilitation groups 12 days after surgery, designated as sets ctr12vs0 and exp12vs0, respectively (**a**). Volcano plots for sets 1vs0 (**b**), ctr12vs0 (**c**), and exp12vs0 (**d**) show the distribution of gene activation in coordinates log_10_(FDR) vs. log_2_(Fold Change). The horizontal dashed line in panels (**b**–**d**) indicates the significance threshold (FDR = 0.05).

One day after surgery, transcriptome analysis of muscle tissue from patients in both the control and experimental groups revealed 1986 genes with significantly down-regulated expression and 593 genes with significantly up-regulated expression compared to preoperative levels (FDR ≤ 0.05) (Figure 4).

At 12 days post-THA, the number of significantly (FDR ≤ 0.05) up-regulated genes compared to preoperative activity in the control group (ctr12vs0) amounted to 546 (Figure 4a). This figure substantially exceeded the number of down-regulated genes in the same group (190). In the experimental rehabilitation group at 12 days post-THA, the count of up-regulated genes was 7-fold higher than in the control group and 15-fold greater than the number of down-regulated genes within the experimental group (exp12vs0, Figure 4a). Figure 4b–d display the distribution of gene activation in log_10_(FDR) vs. log_2_(Fold Change) coordinates (volcano plots) for the 1vs0, ctr12vs0, and exp12vs0 sets.

The differentially expressed gene (DEG) analysis indicates that isometric exercises in the experimental group enhanced gene up-regulation compared to the standard physical rehabilitation protocol in the control group.

Figure S1 shows the 15 genes with the most up- and down-regulated expression levels in the 1vs0, ctr12vs0, and exp12vs0 sets. Among these gene subsets, several unidentified genetic sequences were detected. Significant differences in the composition of these gene subsets preclude their meaningful use for the comparative analysis of ctr12vs0 and exp12vs0 gene sets.

#### 2.2.2. Gene Set Enrichment Analysis

**Figure 5:**
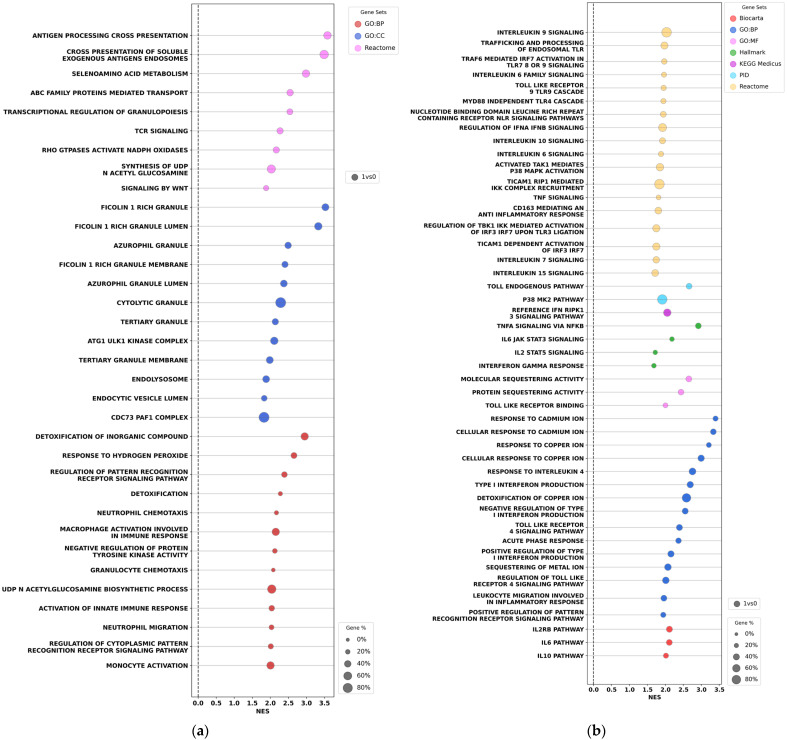
Gene Set Enrichment Analysis (GSEA) dot plots show activation of gene sets associated with the immune response (**a**) and inflammation (**b**) in the rectus femoris 1 day after THA, compared to that at the start of the operation, in both the control and experimental rehabilitation groups. NES—Normalized Enrichment Score. Dot color denotes the different databases used in the analysis. Dot size reflects the percentage of identified genes per gene set.

Gene Set Enrichment Analysis (GSEA) of biological processes and signaling pathways associated with differentially expressed genes revealed significant up-regulation (Normalized Enrichment Score, NES ≥ 1.5) of multiple biological processes, molecular functions, and signaling pathways in muscle biopsies from both the control and experimental rehabilitation groups at 1 day postsurgery, relative to preoperative activities. No significant (NES ≤ −1.5) down-regulation was observed in both groups at this time. Notably, GSEA identified the activation of immune-response-related processes and molecular functions, including monocytic activation, neutrophil migration, neutrophil chemotaxis processes, granulocyte chemotaxis, macrophage activation involved in immune response, response to hydrogen peroxide, and activation of innate immune response (Figure 5a).

On the first postsurgery day, a broad activation of the sets of genes associated with the pro-inflammatory response of muscle tissue was detected, for example, activation of interleukin signaling pathways IL-1, IL-2, IL-4, IL-6, IL-8B, IL-9B, IL-10B, IL-12, IL-13, IL-6 JAK STAT3 signaling, IL-2 STAT3 signaling, response to IL-4, IL-1 induced 7dentition of NFKB, regulation of IFNα and IFNβ signaling, regulation of IFNγ signaling, toll-like receptor (TLR) cascades, regulation of TLR by endogenous ligand, and negative regulation of type 1 IFN production (Figure 5b).

Additionally, GSEA revealed activation of the gene sets of processes related to cell cycle regulation, as well as numerous pathways associated with gene transcription, protein synthesis, and protein degradation (Figures S2–S4). Furthermore, autophagy and apoptosis were activated in muscle tissue. This included both up- and down-regulation of intrinsic apoptotic processes, positive regulation of muscle cell apoptotic processes, and oppositely directed mechanisms of apoptosis stimulation and suppression (Figure S5).

**Figure 6:**
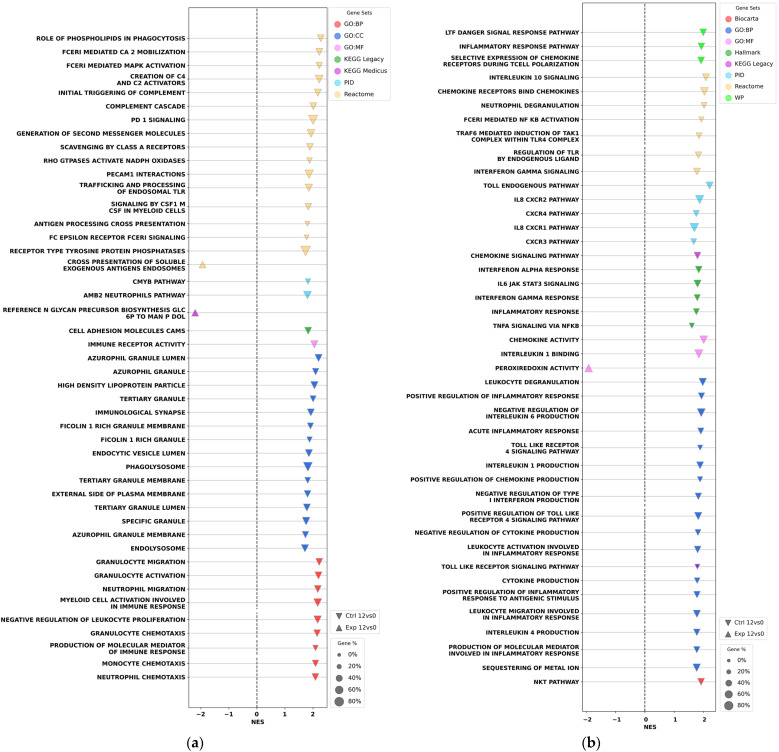
GSEA shows activation of the gene sets associated with the immune response (**a**) and inflammation (**b**) in the rectus femoris of patients in the control rehabilitation group 12 days after THA compared to that at the start of the operation, but not in the experimental rehabilitation group. NES—Normalized Enrichment Score. Dot color denotes the different databases used in the analysis. Dot size reflects the percentage of identified genes per gene set.

GSEA dot plots revealed substantial differences between the experimental and control rehabilitation groups 12 days after THA (Figure 6). In the control group, 12 days post-THA, activation of the gene sets associated with immune response and inflammation persisted similar to the pattern observed 1 day post-surgery (Figure 6a). Specifically, the activation of gene sets of acute-phase cellular immunity was detected, including activation of neutrophils, granulocytes, monocytes, macrophages, and pro-inflammatory response mediated by interleukins (e.g., IL-6, IL-8, IL-1, and others). In contrast, the experimental group showed no significant up-regulation of inflammation- and 8dentity-related processes or signaling pathways at this time point, and down-regulation was detected only for a few isolated processes, such as peroxiredoxin activity (Figure 6b). These findings indicate that the immune response to surgical intervention and associated inflammation persisted in the control group, whereas these processes were resolved after the experimental rehabilitation.

**Figure 7:**
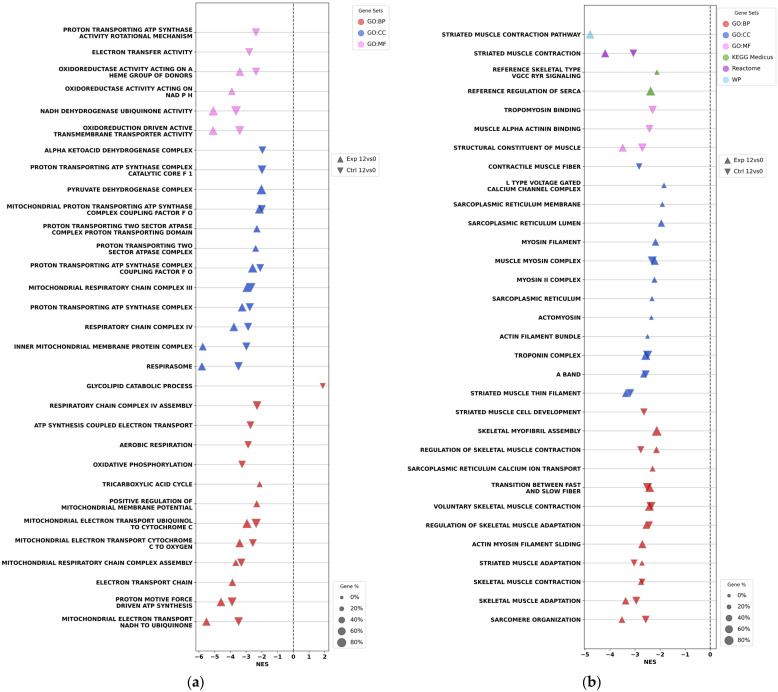
GSEA dot plots show down-regulation of the gene sets associated with the oxidative phosphorylation (**a**) and muscle contraction (**b**) in the rectus femoris of patients in both the control and experimental rehabilitation groups 12 days after THA compared to that at the start of the operation. NES—Normalized Enrichment Score. Dot color denotes the different databases used in the analysis. Dot size reflects the percentage of identified genes per gene set.

GSEA dot plots revealed down-regulation of the gene sets associated with oxidative phosphorylation (Figure 7a) and muscle contraction (Figure 7b) in both the control and experimental groups at the end of early rehabilitation. Specifically, down-regulation was observed for respiratory chain complex III, respiratory chain complex IV, respiratory chain complex assembly, ATP synthesis-coupled electron transport, tricarboxylic acid cycle, and numerous other related processes. Similarly, both rehabilitation groups showed at this time down-regulation of the molecular complexes and processes linked to muscle contraction, including striated muscle contraction, muscle myosin complex, myosin filament, L-type gated calcium channel complex, transition between fast and slow fiber types, skeletal muscle adaptation, sarcomere organization, and other related pathways. Overall, these findings indicate that additional IEs to the standard protocol did not impede the reduction in contractile activity of the rectus femoris in the operated leg during the first two weeks after THA surgery in contrast to the effect on the postoperative immune response and inflammation.

#### 2.2.3. Comparative Analysis of DEG Associated with Selected Biological Processes for the Contrasts 1vs0, ctr12vs0, and exp12vs0

**Figure 8:**
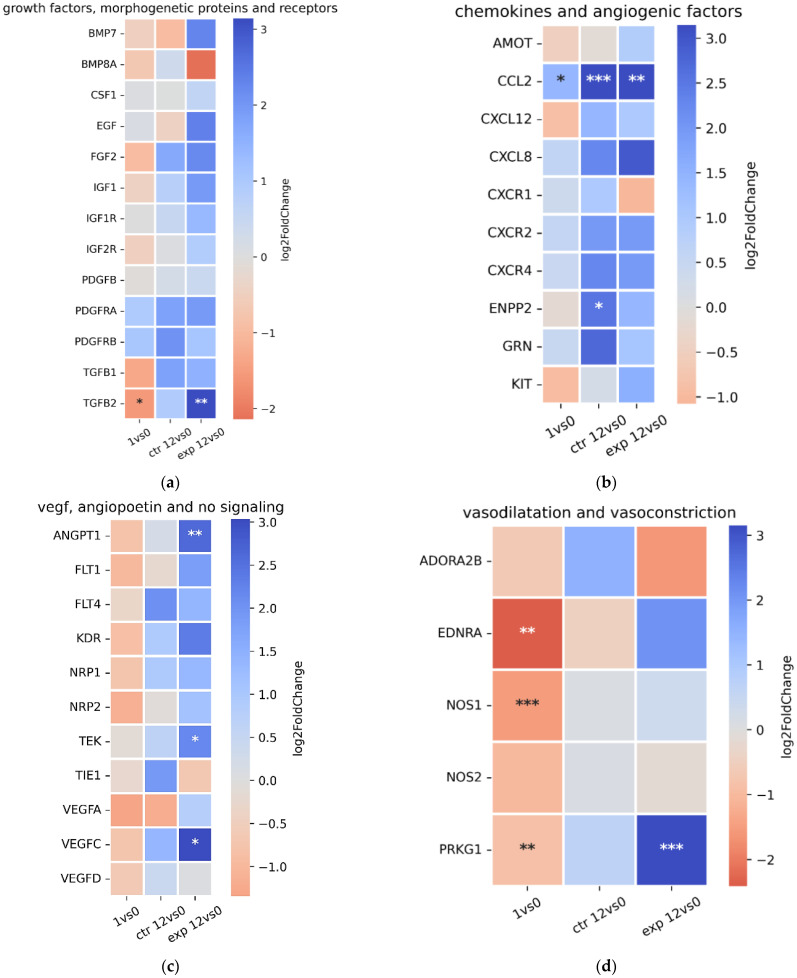
Heatmaps of DEG related to growth factors and morphogenetic proteins (**a**); chemokines and angiogenic factors (**b**); vascular endothelial growth factor (VEGF), angiopoietin, and NO signaling (**c**); and vasodilatation and vasoconstriction processes (**d**) associated with groups 1vs0, ctr12vs0, and exp12vs0. FDR < 0.05, 0.01, and 0.001 for *, **, and ***, respectively.

A comparative analysis of differentially expressed genes for growth factors and morphogenetic proteins (Figure 8a) across the 1vs0, ctr12vs0, and exp12vs0 sets revealed no qualitative differences between the control and experimental rehabilitation groups. However, the experimental group exhibited higher expression levels of most genes compared to the control group. Notably, elevated expression of bone morphogenetic protein 7 (*BMP7*) was observed in the experimental group. *BMP7* is a key regulator of osteogenesis, promoting the differentiation of mesenchymal stem cells into osteoblasts. It also exerts anti-inflammatory and anti-fibrotic effects in various tissues and enhances tissue repair by stimulating cell growth and differentiation [20]. The experimental group also showed significantly increased expression of transforming growth factor beta 2 (*TGFB2*), which is involved in wound healing, angiogenesis, and extracellular matrix formation. Additionally, in the exp12vs0 set, up-regulated expression of the epidermal growth factor (*EGF*) gene was detected, suggesting enhanced wound-healing processes. Conversely, the experimental rehabilitation group displayed down-regulated expression of bone morphogenetic protein 8A (*BMP8A*), which plays an important role in osteogenesis, chondrogenesis, lipid metabolism, and energy substrate availability for muscle tissue [21]. The reduced *BMP8A* expression may indicate enhanced muscle atrophy [22].

For the category of chemokines (Figure 8b), no qualitative differences were observed between the control and experimental rehabilitation groups. However, the exp12vs0 set showed elevated expression relative to ctr12vs0 for *AMOT* (angiomotin protein), which regulates endothelial cell migration, adhesion, cell polarity, and angiogenesis, and *KIT* (tyrosine kinase receptor), which is vital for multiple cellular processes, including cell motility and adhesion, and is particularly essential for mast cell survival and function. Conversely, reduced expression in the exp12vs0 set compared to ctr12vs0 was observed for the C-X-C motif chemokine receptor 1 (*CXCR1)* gene, which encodes a GPCR receptor. This receptor plays a key role in immune responses and inflammation by binding to chemokines, notably IL-8. The down-regulation of *CXCR1* may indicate a more 12ffecttive resolution of inflammation in the experimental group relative to the control. Additionally, higher expression levels of the ectonucleotide pyrophosphotase/phosphodiesterase 2 (*ENPP2*) and the granulin precursor (*GRN*) in ctr12vs0 compared to exp12vs0 may suggest more severe inflammation in the control group versus the experimental rehabilitation group. The ENPP2 protein acts as a phosphodiesterase and lysophospholipase D, generating lysophosphatidic acid (LPA) signals via G protein-coupled receptors. It influences cell motility, proliferation, survival, inflammation, and angiogenesis [23]. The progranulin protein, encoded by the *GRN* gene, plays a crucial role in cell growth and survival, as well as in regulating inflammation and wound healing [24].

Differences between the ctr12vs0 and exp12vs0 sets were also observed for genes related to the vascular endothelial growth factor (VEGF), angiopoietin, and NO signaling pathways (Figure 8c). In the exp12vs0 group, a higher number of DEGs was detected compared to ctr12vs0 for the following genes: angiopoietin-1 (*ANGPT1)*, Fms-related receptor tyrosine kinase 1 (*FLT1*), kinase insert domain receptor (*KDR*), neuropilin-2 (*NRP2*), receptor tyrosine kinase (*TEK*), vascular endothelial growth factor A (*VEGFA*), and vascular endothelial growth factor A (*VEGFC*). *ANGPT1* encodes angiopoietin-1, a ligand for the Tie2 receptor and a key regulator of blood vessel formation (angiogenesis) and maturation. *ANGPT1* can inhibit the expression of genes associated with inflammation and reduce immune cell recruitment to blood vessels. It also promotes proliferation, migration, and differentiation of skeletal muscle cells (myoblasts), thereby supporting muscle repair after injury [25]. *KDR* encodes the protein of receptor VEGFR2, a central regulator of angiogenesis and a critical factor in wound healing. The binding of VEGF proteins to KDR triggers intracellular signaling pathways, including MAPK and PI3K-AKT [26]. *FLT1* encodes VEGFR1, the receptor for VEGFA. *FLT1* can act as both a positive and negative regulator of angiogenesis and is essential for endothelial cell growth, migration, and differentiation, as well as for angiogenesis and vasculogenesis. *NRP2* encodes neuropilin-2, a protein involved in axon guidance, angiogenesis, lymphangiogenesis, and immune responses. Neuropilin-2 binds ligands such as semaphorins and VEGFs. Receptor tyrosine kinase (*TIE2*) encodes the TEK receptor, predominantly active in endothelial cells. TEK is essential for angiogenesis and hematopoiesis and mediates cross-talk between endothelial cells and surrounding smooth muscle cells [27]. *VEGFA* encodes protein VEGFA, a major angiogenic regulator that stimulates endothelial cell proliferation, migration, and survival, thus supporting wound healing. VEGFA also acts as a chemoattractant and mitogen for osteoblasts, promoting bone formation [28]. *VEGFC* encodes VEGFC, a key regulator of lymphangiogenesis (formation of new lymphatic vessels) and angiogenesis. VEGFC also contributes to inflammatory responses, neural development, and maintenance of the bone marrow perivascular niche [29]. Conversely, the ctr12vs0 group showed higher expression of Fms-related receptor tyrosine kinase 4 (*FLT4)* compared to exp12vs0. *FLT4* encodes VEGFR3, which is activated by VEGFC and VEGFD and plays a critical role in lymphatic vessel formation and function, as well as in angiogenesis [30]. Overall, these findings suggest that the experimental group exhibited enhanced 13dentition of angiogenesis and lymphangiogenesis, as well as greater resolution of inflammation, compared to the control group during the early rehabilitation phase.

Figure 8d presents a heatmap of selected genes from the 1vs0, ctr12vs0, and exp12vs0 sets involved in vasoconstriction, vasodilation, and angiogenesis. Noteworthy is the down-regulated expression of gene adenosine A2b receptor (*ADORA2B)* in exp12vs0 compared to the up-regulation of this gene in ctr12vs0, exceeding the expression of endothelin receptor type 1 (*EDNRA*) and protein kinase cGMP-dependent 1 (*PRKG1*) genes in exp12vs0 in comparison to ctr12vs0. *ADORA2B* encodes the adenosine A2B receptor (a GPCR receptor) involved in angiogenesis, metabolism, and inflammatory responses to non-antigenic stimuli. This receptor acts as a sensor of hypoxia and metabolic stress, promoting angiogenesis via VEGF production, cytokine release, and PI3K/AKT pathway activation [31,32]. The down-regulation of *ADORA2B* in the exp12vs0 set may indicate suppression of inflammatory processes. *EDNRA* encodes endothelin receptor type A (ETA), which mediates potent vasoconstrictive effects upon binding with endothelin-1 (ET-1). The elevated *EDNRA* expression in exp12vs0 relative to ctr12vs0 may reflect the enhanced potential of vasoconstriction [33]. Notably, *EDNRA* was strongly up-regulated one day postsurgery, suggesting suppression of vasoconstriction prior to rehabilitation. *PRKG1* encodes cGMP-dependent protein kinase 1 (PKG1), a key mediator of NO and cGMP signaling. PKG1 promotes smooth muscle relaxation, leading to vasodilation and blood pressure reduction, and inhibits platelet activation to prevent excessive coagulation [34]. *NOS1* and *NOS2* encode neuronal nitric oxide synthase (nNOS) and inducible nitric oxide synthase (iNOS), respectively. These enzymes catalyze NO production from L-arginine. NO functions as a signaling molecule that induces smooth muscle cell relaxation (vasodilation) [35]. The expression levels of *NOS1* and *NOS2* were comparable between ctr12vs0 and exp12vs0 groups and significantly exceeded those in the 1vs0 group. The data presented in Figure 8d suggest that the experimental group exhibited enhanced suppression of inflammation and activation of the genes associated with both vasoconstrictive and vasodilative processes in the rectus femoris of the operated limb compared to the control group.

## 3. Discussion

The analysis of rehabilitation programs following THA points to problems in assessing their efficacy [36,37], and identifying molecular indicators for such an assessment is a pressing task.

It is well established that various blood parameters exceed normal reference ranges following THA and subsequently return to baseline levels. In our study, markers of muscle injury—including serum myoglobin and CPK concentrations—exhibited a marked increase on the first postoperative day. However, by 12 days post-THA, these values had returned to normal ranges in both the control and experimental groups. These findings are consistent with the literature data [38,39,40]. The rapid normalization of muscle injury biomarkers following THA limits their utility as indicators of early rehabilitation efficacy.

Total white blood cell count and neutrophil count exhibited a moderate increase on the first day after THA, exceeding the upper limits of normal values, and then declined to values approaching the upper limit at day 12 postsurgery in both the control and experimental groups. These observations align with the published data [41]. In the experimental group, white blood cell and neutrophil counts returned to normal ranges in all patients by day 12 post-THA. In the control group, normalization occurred in only half of the patients; however, this difference between groups was not statistically significant according to a Fisher’s exact test (*p* = 0.43; *p* >> 0.05). Mean ESR showed no substantial change from preoperative levels at both 1 and 12 days post-THA in either group, consistent with the existing literature [42,43]. One exception was a single patient in the experimental group who exhibited a marked ESR increase on day 12 due to an acute respiratory viral infection. These results indicate that ESR is not a promising marker for evaluating the efficacy of early rehabilitation protocols following THA. CRP levels showed a significant rise on the first postoperative day, followed by a gradual decline in both the control and experimental groups. However, by day 12 post-THA, CRP levels remained significantly above normal reference ranges in both groups, with no statistically significant difference between them according to a Mann–Whitney test. The prolonged recovery kinetics of CRP following THA preclude its use as a reliable indicator for assessing the efficacy of early rehabilitation interventions.

As is well known, muscle tissue damage during THA triggers a systemic pro-inflammatory response, followed by an anti-inflammatory phase and subsequent regenerative processes [44,45]. In our study, we observed a significant increase in serum concentrations of IL-6 and IL-8 one day post-THA. IL-6 levels rose to 144 pg/mL (upper normal limit: 8 pg/mL); IL-8 levels reached 29 pg/mL (upper normal limit: 10 pg/mL). IL-1β concentrations increased within normal limits one day postsurgery in most patients, with one exception in the control group where levels exceeded the reference range. By day 12 post-THA, IL-6, IL-8, and IL-1β concentrations had returned to normal ranges in all patients in the experimental group. In the control group, only a subset of patients showed cytokine normalization, with comparable recovery kinetics across groups. These changes in IL-6, IL-8, and IL-1β levels following THA are consistent with reports that IL-6 plasma concentrations may rise to 30–430 pg/mL within the first 1–3 days postsurgery, returning to normal ranges within the next 2 weeks [42,43]. Significant elevation of IL-8 concentration in blood within the first 12–24 h post-THA was noted also in [46]. Antonov A.A. et al. [41] revealed significantly increased IL-6 and IL-8 levels on days 1–3 postsurgery, along with a moderate rise in IL-1 within the first 24 h after THA. A marked increase was demonstrated in IL-6 blood levels during the first 1–3 days post-THA, followed by a decline and normalization within 2 weeks [43]. The question arises is whether it is possible to use these changes in interleukin levels to compare the effectiveness of control and experimental early rehabilitation methods after THA in groups with small numbers of patients. To assess the significance of the difference between the rehabilitation groups, we analyzed whether interleukin levels were within normal ranges in patients of the groups on day 12 after surgery. In the experimental group, all patients (*n* = 4) showed IL-6, IL-8, and IL-1β values within normal limits (12 within normal and 0 above normal) on day 12 postsurgery. In contrast, the control group (*n* = 4) exhibited 5 values above normal and 7 within the normal range. According to a Fisher’s exact test, this difference between the groups was statistically significant (*p* = 0.037; *p* < 0.05). This finding demonstrates that even for small groups, reliable group differences can be detected when using a composite of independent blood parameters with similar post-THA normalization kinetics. When expanding this composite index to include leukocytes and 14dentifphils, which show comparable patterns of normalization with interleukins IL-6, IL-8, and IL-1β, the results 12 days after THA were as follows. In the experimental group, all 20 values were within normal limits (20 within normal limits; 0 above normal), and in the control group, 11 values were within normal limits and 9 were above normal. A Fisher’s exact test yielded *p* = 0.0012, indicating a high significant difference between groups. These results suggest that the experimental early rehabilitation protocol accelerated the resolution of the systemic inflammation induced by THA. The faster resolution of systemic inflammation via IEs can be interpreted as an indicator of accelerated overall postoperative recovery and thus as evidence of enhanced rehabilitation efficacy.

Transcriptomic analysis of rectus femoris biopsies from the operated leg was employed to assess tissue recovery near the surgical area following THA. According to GSEA, one day postsurgery, we observed the activation of gene sets involved in the pro-inflammatory response of muscle tissue. These included IL signaling pathways IL-1, IL-2, IL-4, IL-6, IL-8B, IL-9B, IL-10B, IL-12, IL-13, IL-6 JAK-STAT3 signaling, IL-2 STAT3 signaling, response to IL-4, IL-1-induced NF-κB activation, regulation of IFNα and IFNβ signaling, regulation of IFNγ signaling, TLR cascades, TLR regulation by endogenous ligand, and negative regulation of type I IFN production (Figure 5b). Twelve days post-THA, the control group still exhibited the activation of gene sets associated with immune response and inflammation—similar to patterns observed one day after surgery (Figure 6a,b). Specifically, acute-phase cellular immunity was activated, including activation of neutrophils, granulocytes, monocytes, macrophages, and pro-inflammatory responses linked to interleukins (e.g., IL-6, IL-8, and IL-1). In contrast, the experimental group showed no significant activation or suppression of these processes by day 12 (Figure 6b). These findings indicate that inflammation persisted in the control group, but the inflammatory response was resolved in the experimental group by day 12 postsurgery. Notably, the resolution of general inflammation in the body, as revealed by ELISA analysis of blood interleukins 12 days post-THA, is consistent with the resolution of local inflammation in the muscle tissue of the operated leg, as revealed by transcriptome analysis.

In contrast to inflammation-related pathways and processes, GSEA dot plots reveal down-regulation of the gene sets associated with oxidative phosphorylation (Figure 7a) and muscle contraction (Figure 7b), both in the control and experimental rehabilitation groups 12 days after THA compared to preoperational activities. These findings indicate that during the early rehabilitation phase, the additional IEs do not activate the transcriptomic patterns related to energy metabolism and contractile processes in muscle tissue. This outcome is consistent with clinical expectations, as patient mobility remains limited in the first two weeks following THA in both groups—a factor that likely contributes to the observed down-regulation of these processes.

Notably, in the experimental rehabilitation group 12 days post-THA, 3989 DEGs were up-regulated, and 290 were down-regulated. However, GSEA analysis revealed no statistically significant up-regulation of the associated sets of genes at that time. A similar pattern was observed in both the control and experimental groups one day postsurgery: 593 DEGs were up-regulated, and 1986 were down-regulated, yet GSEA did not detect significant down-regulation of the associated sets of genes. This apparent discrepancy is not inherently contradictory, but its biological significance requires further investigation.

Heatmap analysis of DEG associated with growth factors and morphogenetic proteins, chemokines, angiogenic factors, VEGF, angiopoietin, NO signaling pathways, and vasodilation and vasoconstriction processes revealed differences between the experimental and control rehabilitation groups for several of these gene sets. For example, genes for growth factors, morphogenetic proteins, and their receptors showed higher activation in the experimental group compared to the control, with only a few genes exhibiting lower activation. Overall, these transcriptomic changes suggest enhanced regenerative processes and the establishment of conditions conducive to tissue repair. This may involve the down-regulation of specific genes—such as *BMP8A* and the chemokine receptor *CXCR1*—potentially contributing to suppression of the immune response in muscle tissue [47]. DEG heatmaps for VEGF, angiopoietin, and NO signaling pathways indicate that the experimental group exhibited up-regulated angiogenesis and lymphangiogenesis (evidenced by *FLT4*, *VEGFA*, *VEGFC*, *ANGPT1*, *NRP2*, *TEK*, and *ADORA2B*) and enhanced suppression of inflammation (via *ANGPT1* and *ADORA2B*) compared to the control group during the early rehabilitation phase. Analysis of DEG further revealed an increased activity of genes associated with vasoconstriction (*EDNRA* gene) and vasodilation (*PRKG1* gene) in the muscle tissue of patients in the experimental group relative to the control group. These findings suggest that IEs promote regenerative processes in muscle tissue by modulating vascular responses.

To perform transcriptomic analysis, we used a muscle tissue biopsy, which is an invasive procedure, but it provides valuable data on biological processes and molecular functions for research purposes. For routine clinical monitoring of rehabilitation efficacy, the use of a “composite index” of interleukins IL-6, IL-8, and IL-1β appears more practical and less invasive.

Our work is a pilot study and includes a limited group of female patients in a narrow age range of 73–77 years. We believe that, on the one hand, this was an advantage and provided reliable differences between the control and experimental groups in the “healing signatures” of local damage by muscle transcriptome data and by the cytokine “composite index” in the blood. On the other hand, this set of patients is a limitation and does not allow extrapolating the results to patients of a different gender and age. These limitations require additional study for patients of a different gender and age.

## 4. Materials and Methods

### 4.1. Study Design

The study protocol and the experimental rehabilitation methodology were approved by the Local Ethics Committee (LEC) of Pushchino Scientific Centre Hospital of the Russian Academy of Sciences (RAS). The research was conducted in accordance with current standards of Good Clinical Practice and the principles of the Declaration of Helsinki. All patients participating in the program provided written informed consent prior to inclusion in the study. The informed consent form was approved by the LEC of Pushchino Scientific Centre Hospital, RAS.

Eight female patients (Caucasion) aged 73–77 years underwent THA using a Smith&Nephew cemented prosthetic system with an anterolateral Müller approach (ICD codes: M16.0, M16.1). The implant featured a metal-on-polyethylene bearing surface. THA was performed due to severe clinical manifestations of stage III coxarthrosis, as classified by N.S. Kosinskaya. Exclusion criteria included the presence of acute or decompensated somatic conditions, as well as those with any severe comorbidities that, in the physician’s judgement, could hinder study participation. Patient exclusion due to failure to comply with protocol requirements was anticipated but not implemented, and all patients fully completed the rehabilitation programs. All THA procedures were performed under general anesthesia using standard anesthetic protocols. Patients were allocated to two groups sequentially (without formal randomization) as they were enrolled according to the dates of their hospitalization. The control group (*n* = 4) followed standard IE protocols in accordance with the Russian Ministry of Health’s national clinical guidelines “Coxarthrosis” [48]. Patients of the experimental group (*n* = 4) performed a novel rehabilitation regimen incorporating additional IEs, as described in our patent [49]. Postoperative care for both groups was managed by a single orthopedic surgeon according to established clinical protocols.

Rehabilitation was initiated on the first postoperative day following THA and continued for 12 consecutive days. Each patient underwent two daily sessions, with session duration ranging from 15 min to 1 h depending on the assigned rehabilitation protocol. The IE protocol for the patients of the experimental and control groups is described below.

Thomas test isometric exercises in the experimental group were performed in the supine position on postoperative days 1–2: 5–7 s per exercise, 4–5 exercises, once daily; on days 3–7: 8–9 s per exercise, 6–7 exercises, twice daily; on days 8–12: 10–12 s per exercise, 7–8 exercises, twice daily. The patients in the control group performed the same exercises for 5 s each, 5–7 exercises, twice daily, all days.

IEs for gluteal muscles in the experimental group were performed in the supine position, 5 exercises once daily, with the following duration per exercise: day 1: 15–20 s; day 2: 20–25 s; day 3: 35–40 s; day 4: 50–60 s; day 5: 65–75 s; days 6–7: 80–90 s; and days 8–12: 2 min. The patients in the control group performed the same exercises: 8–10 exercises, 7 s each, twice daily, all days.

IEs for anterior thigh muscles of the operated leg in the experimental group were performed in the supine position, 5 exercises once daily, with the following duration per exercise: days 1–3: 15–20 s; days 4–5: 25–30 s; day 6: 40–50 s; day 7: 55–65 s; day 8: 70–80 s; and days 9–12: 85–95 s. The patients in the control group performed the same exercises: 8–10 exercises, 7 s each, twice daily, all days.

IEs for posterior thigh muscles of the operated leg in the experimental group were performed in the supine position once daily, following the schedule described above for gluteal muscles. The patients in the control group performed the same exercises: 8–10 exercises, 7 s each, twice daily, all days.

IEs for anterior thigh muscles of the non-operated leg in the experimental group were performed with resistance provided by a rehabilitation specialist’s hands during hip and knee flexion in the supine position, 5 exercises once daily, with the following duration per exercise: day 1: 15–20 s; day 2: 25–30 s; day 3: 35–40 s; days 4–6: 45–50 s; days 7–8: 55–65 s; and days 9–10: 85–95 s. The patients in the control group did not perform these exercises.

IEs for abductor muscles of the non-operated leg (flexed at hip and knee joints) in the experimental group were performed with resistance provided by a rehabilitation specialist’s hands during abduction to the side, in the supine position, 5 exercises once daily, with the following duration per exercise: day 2: 10–15 s; day 3: 25–30 s; day 4: 40–45 s; and days 5–6: 55–65 s. The patients in the control group did not perform these 17dentifyes.

IEs for adductor muscles of the non-operated leg (flexed at hip and knee joints) in the experimental group were performed with resistance provided by a rehabilitation specialist’s hands during movement toward the operated leg, in the supine position once daily, following the schedule described above for abductor muscles. The patients in the control group did not perform these exercises.

IEs for thigh muscles of the non-operated leg during knee extension in the experimental group were performed in a semi-sitting position with legs hanging down, with the foot in dorsiflexion during the exercise, 5 exercises once daily, with the following duration per exercise: day 4: 25–30 s; day 5: 40–50 s; day 6: 55–65 s; and day 7: 70–80 s. The patients in the control group performed the same exercises: 8–10 exercises, 5–7 s each, twice daily, on postoperative days 4–12.

IEs for thigh muscles of the operated leg during knee extension in the experimental group were performed in a semi-sitting position with legs hanging down, with the foot in dorsiflexion during the exercise, 5 exercises once daily, with the following duration per exercise: day 4: 10–15 s; day 5: 25–30 s; days 6–8: 40–50 s; and days 9–12: 60–70 s. The same exercises in the control group were performed: 8–10 exercises, 5–7 s each, twice daily, on postoperative days 4–12.

IEs for anterior thigh muscles of both legs in the control group were performed in the supine position, 5 exercises, 5–7 s per exercise, twice daily, on postoperative days 4–12. The patients in the experimental group did not perform these exercises.

Thus, for the experimental group the time performing IEs was increased by 6–10 times, and additional IEs were added.

According to the Lequesne scale analysis, most patients exhibited severe to very severe functional limitation prior to surgery [9,48]. Harris Hip Score assessments yielded values below 70 points, which is classified as a poor outcome. Performance on the «Timed Up and Go» (TUG) test revealed gait and balance impairments in all patients, with most requiring more than 14 s to complete the task. In the 10-metre walk test, patients in both groups took longer than 20 s to finish. All participants required assistive devices (e.g., walkers or canes) to complete the walking tests. These findings indicate a high risk of falls and slow walking speed. Preoperative assessment of patient condition and functional activity—using the Lequesne and Harris scales, the TUG test, and the 10-metre walk test—revealed no statistically significant differences between the groups (*p* > 0.05) (Table S3).

Overall, patients in the two study groups did not differ with respect to functional test results, comorbidities, blood parameters within normal ranges prior to surgery, and surgical intervention severity, as assessed by postoperative day 1 blood parameters (*p* > 0.05). No adverse effects were observed during all rehabilitation periods in both groups.

### 4.2. Clinical Laboratory Diagnostics

For each patient, the following laboratory tests were performed: complete blood count with differential leukocyte count, ESR, CRP level, myoglobin, AST, LDH, CPK, and serum cytokine measurements (IL-1β, IL-6, IL-8, and TNFα). Standard clinical laboratory methods were used. Blood samples were collected from the cubital vein after an overnight fast 1 day before surgery, on the first postoperative day, and 12 days after surgery. Serum concentrations of IL-1β, IL-6, IL-8, and TNFα were measured using an enzyme-linked immunosorbent assay (ELISA) with test kits from JSC «Vector-Best» (Novosibirsk, Russia).

### 4.3. Percutaneous Muscle Biopsies

All enrolled patients underwent muscle tissue biopsies three times under fasting conditions: at the start of operation, 1 day postoperation, and 12 days post-operation. The second and third biopsies were performed under local anesthesia with 2% lidocaine. Biopsy samples (30 mg each) were collected from the rectus femoris using a disposable Multicore soft-tissue biopsy needle (14G, 20 cm length) at a depth of 2–3 cm. All specimens were immediately placed in EverFresh RNA lysis buffer and stored at −28 °C for subsequent RNA sequencing analysis.

### 4.4. Isolation and Sequencing of Muscle Tissue RNA

RNA was extracted from frozen tissue samples (~30 mg) using a HiPure Viral RNA Kit (Magen, Guangzhou, China). RNA concentration was quantified using a fluorometer (Qubit 4.0; Thermo Fisher Scientific, Waltham, Massachussets, USA). Complementary DNA (cDNA) synthesis was performed using previously isolated mRNA and a Mint kit (Evrogen, Moscow, Russia). The procedure followed the manufacturer’s protocol, including the addition of IP-solution to enhance synthesis efficiency. Subsequently, the synthesized cDNA was amplified using an Encyclo Plus PCR Kit (Evrogen, Moscow, Russia) with universal primers. To verify amplification success, the resulting products were visualized via horizontal agarose gel electrophoresis.

For library preparation, 200 ng of cDNA from each sample was used. Sequencing libraries were constructed from the obtained amplicons using a Ligation Sequencing Kit (SQK-LSK109, Oxford Nanopore Technologies, Oxford, UK), following the manufacturer’s protocols. During the DNA end-preparation step, End Prep Mix was added to the samples. The mixture was thoroughly mixed by pipetting, and droplets on the tube walls were removed via brief centrifugation. The reaction was carried out in a thermal cycler. After incubation, the mixture was purified using magnetic beads (VAHTS DNA Clean Beads, Vazyme, Nanjing, China). The DNA was eluted in 10 µL of Milli-Q water, and its concentration was measured using a Qubit 4 fluorometer (Thermo Fisher Scientific, Waltham, Massachussets, USA) with a QuDye HS Assay Kit (Lumiprobe, Moscow, Russia). cDNA libraries were multiplexed for sequencing via native barcoding using a PCR Barcoding Expansion 1–96 kit (EXP-PBC096, Oxford Nanopore Technologies, UK), according to the manufacturer’s protocol. Samples were purified with VAHTS DNA Clean Beads (Vazyme, Nanjing, China). The concentration of the pooled samples was measured on the Qubit 4. Following ligation, the mixture was cleaned with magnetic beads, washed with SFB (by removal from the magnet and resuspending the beads), and eluted in EB buffer. The final library concentration was verified using the Qubit 4, after which the library was loaded onto the sequencing flow cell. The prepared DNA library was sequenced on an Oxford Nanopore PromethION platform using a FLO-PRO112D flow cell, following the standard protocol and MinKNOW software (v. 24.11.10; Oxford Nanopore, Oxford, UK).

The raw sequencing data is available in the Gene Expression Omnibus (GEO) database using identifier GSE315228 or via direct link https://www.ncbi.nlm.nih.gov/geo/query/acc.cgi?acc=GSE315228 (submitted on 30 December 2025).

### 4.5. Bioinformatics Analysis

The quality of raw sequencing data was assessed using NanoPlot software (v. 1.43.0; https://github.com/wdecoster/NanoPlot (accessed on 12 January 2025)). Basecalling and adapter sequence removal were performed with dorado software (v. 0.8.3; https://github.com/nanoporetech/dorado (accessed on 13 January 2025)). For the alignment and annotation of sequencing data, we used the human reference genome assembly and annotation file from the Ensembl database (version 113, GRCh38.p14; https://www.ensembl.org/Homo_sapiens/Info/Index (accessed on 13 January 2025)). The reference genome was indexed using minimap2 (v. 2.28; https://github.com/lh3/minimap2/?tab=readme-ov-file#map-long-splice (accessed on 13 January 2025)), followed by read counting with featureCounts from the subread package (v. 2.0.8; https://github.com/ShiLab-Bioinformatics/subread (accessed on 13 January 2025)). Differential gene expression analysis was conducted using DESeq2 software (v. 1.38.3) in the R programming language (v. 4.2.2; accessed on 13 January 2025) [50].

### 4.6. Differential Gene Expression Analysis

Differential gene expression analysis was performed across three comparison groups involving two experimental conditions—the control and experimental rehabilitation groups.

We compared sequencing results from muscle tissue biopsies, collected 12 days post-THA versus preoperative muscle biopsy samples, and sequencing data from muscle biopsies of both the experimental and control patient groups collectively, obtained 1 day postoperation, relative to preoperative samples from the same patients.

### 4.7. Gene Set Enrichment Analysis

GSEA was performed using gene sets from the Molecular Signature Database (MsigDB) and the GSEApy package (v. 1.1.6) for the Python programming language (v. 3.11.7; accessed on 20 February 2025) [51].

### 4.8. Statistical Analysis

The significance of differences between the control and experimental groups in systemic inflammation markers—including CRP, white blood cell count, neutrophil count, interleukin levels (IL-1β, IL-6, IL-8), and ESR—was assessed at three time points: preoperatively, 1 day postoperation, and 12 days postoperation. Statistical analysis was performed using a Mann–Whitney U test, and a Fisher’s exact test was applied to evaluate the proportion of patients whose values returned to the normal range. Muscle injury biomarkers (CPK, myoglobin, AST, and LDH) were analyzed using a Mann–Whitney U test. Differences in functional test scores (preoperatively) and dynamometric assessments of lower limb muscle strength (during rehabilitation) between the control and experimental rehabilitation groups were also evaluated using a Mann–Whitney U test. All statistical analyses were conducted using IBM SPSS Statistics software (version 27). The statistical significance of DEG was assessed using a Wald test with Benjamini–Hochberg correction for multiple testing (FDR) [52]. For GSEA results, statistical significance was evaluated using a 20dentiftion test, followed by FDR correction. The significance of functional analysis results was assessed using a Fisher’s exact test, with subsequent FDR correction for multiple testing. Findings were considered statistically significant at FDR ≤ 0.05.

## 5. Conclusions

The findings indicate that the experimental early rehabilitation protocol following THA, which incorporates additional IEs beyond the standard regimen, accelerates the resolution of both systemic inflammation and local inflammatory processes in the muscle tissue near the surgical area. The additional IEs promote activation of the transcription of proteins associated with angiogenesis, lymphangiogenesis, vasodilation, and vasoconstriction in the rectus femoris biopsies of the operated leg. However, they does not affect the suppression of the activity of gene sets associated with oxidative phosphorylation and muscle contraction during the early postoperative period after THA. Overall, these results suggest that IEs enhance the efficacy of early rehabilitation following THA. Furthermore, our data demonstrate that systemic inflammation markers such as concentrations of interleukins IL-6, IL-8, and IL-1β in the blood may collectively serve as molecular indicators of early rehabilitation protocol effectiveness after THA. Additionally, transcriptomic analysis of the rectus femoris muscle in the operated leg enables the identifycation of molecular markers of regenerative processes in the perisurgical muscle tissue. Involving patients of different genders and ages and increasing the size of rehabilitation groups will be a direction for future research to ensure the validity and applicability of the obtained results.

## 6. Patents

Maksimova, E.A.; Akatov, V.S.; Shevchenko, V.I.; Senotov, A.S.; Krasnov K.S. A method of early rehabilitation of patients after hip arthroplasty, 5 November 2025. Patent RU 2850112. Byull. Izobret. 2025, 31.

## Data Availability

The data presented in this study are available in the Supplementary Materials. Raw sequencing data is available in the Gene Expression Omnibus (GEO) database using identifier GSE315228 or via direct link https://www.ncbi.nlm.nih.gov/geo/query/acc.cgi?acc=GSE315228 (submitted on 30 December 2025).

## Supplementary Materials

The following supporting information can be downloaded at: https://www.mdpi.com/article/10.3390/ijms27031389/s1.

## Abbreviations

IEs	Isometric exercises
THA	Total hip arthroplasty
OA	Osteoarthritis
DALYs	Disability-adjusted life years
CRP	C-reactive protein
ESR	Erythrocyte sedimentation rate
GSEA	Gene Set Enrichment Analysis
DEG	Differentially expressed gene
NO	Nitric oxide
AST	Aspartate aminotransferase
LDH	Lactate dehydrogenase
CPK	Creatine phosphokinase
PCA	Principal component analysis
PC	Principal component
NES	Normalized Enrichment Score
TLR	Toll-like receptor
BMP7	Bone morphogenetic protein 7
TGFB2	Transforming growth factor beta 2
BMP8A	Bone morphogenetic protein 8A
EGF	Epidermal growth factor
AMOT	Angiomotin protein
KIT	Tyrosine kinase receptor
CXCR1	C-X-C motif chemokine receptor 1
ENPP2	Ectonucleotide pyrophosphotase/phosphodiesterase 2
GRN	Granulin precursor
LPA	Lysophosphatidic acid
VEGF	Vascular endothelial growth factor
ANGPT1	Angiopoietin-1
FLT1	Fms-related receptor tyrosine kinase 1
KDR	Kinase insert domain receptor
NRP2	Neuropilin-2
TEK	Receptor tyrosine kinase
TIE2	Receptor tyrosine kinase
ETA	Endothelin receptor type A
ADORA2B	Adenosine A2b receptor
EDNRA	Endothelin receptor type 1
PRKG1	Protein kinase cGMP-dependent 1
nNOS	Neuronal nitric oxide synthase
LEC	Local Ethics Committee
RAS	Russian Academy of Sciences
