# Supplementary material for: Molecular Indicators of Isometric Exercise Efficacy in Early Rehabilitation of Older Adults After Total Hip Arthroplasty
Source: Int J Mol Sci. 2026 Jan 30;27(3):1389. doi: 10.3390/ijms27031389 (PMC12898363; doi:10.3390/ijms27031389)
Supplement: Supplementary file 1 [file ijms-27-01389-s001.zip › Supplement Material.pdf]

Table S1. Clinical blood test results of patients in the control and experimental rehabilitation groups 1 day before surgery (0), 1 day (1) and 12 days after THA.

**Blood parameters**	**Patient 1, control group**			**Patient 2, experimental group**			**Patient 3, experimental group**			**Patient 4, experimental group**			**Units of measure-ment**	**Reference ranges**
	0 day	1 day	12	0 day	1 day	12 days	0 day	1 day	12 days	0 day	1 day	12 days		
White blood cells	7,0	11,2	11,5	6,3	14,4	6,8	3,2	7,6	6,5	4,9	16,0	8,9	10^9 /l	4,0 – 9,0
Red blood cells	3,79	3,53	3,17	4,42	3,29	2,77	4,19	3,09	2,73	4,8	4,31	3,2	10^{12} /l	3,90 – 5,00
Hemoglobin	98	92	92	126	98	80	127	94	90	137	113	88	g/l	110 – 160
Hematocrit	29,5	27,5	26,4	37,4	27,7	23,8	37,4	26,8	24,3	41,6	34,5	25,9	%	36 - 48
Platelets	382	291	577	292	249	454	204	230	399	290	235	441	10^9 /l	150 – 400
Band cells	1	3	0	1	3	1	1	4	0	2	4	1	%	1 – 5
Segmented neutrophils	4,26	8,11	8,40	2,72	11,91	3,94	1,39	5,62	4,47	2,84	13,18	6,04	10^9 /l	1,9 – 6,6
Eosinophils	3	1	0	1	1	1	2	1	0	1	1	2	%	1 - 5
Basophils	0	0	0	0	0	0	0	0	0	0	0	0	%	0 – 1
Lymphocytes	29,9	15,3	22,9	46	8,5	33,9	43,4	13,7	25,3	32,0	7,8	21,0	%	19 - 37
Monocytes	5,2	7,3	4,1	8,8	3,8	6,2	10,3	5,4	6,0	7,1	4,8	8,1	%	3 - 11
ESR	25	21	3	29	21	4	3	4	6	20	21	67,9	mm/h	1 -30

Blood parameters	Patient 5, control group			Patient 6, control group			Patient 7, experimental group			Patient 8, control group			Units of measurement	Reference ranges
	0 day	1 day	12 days	0 day	1 day	12 days	0 day	1 day	12 days	0 day	1 day	12 days		
White blood cells	5,9	14,0	8,9	6,9	15,0	8,0	6,5	14,7	7,8	4,9	11,5	9,3	10^9 /l	4,0 – 9,0
Red blood cells	5,89	4,49	3,59	5,16	4,0	2,97	4,69	3,76	3,29	4,0	2,68	2,89	10^{12} /l	3,90 – 5,00
Hemoglobin	151	117	93	141	117	91	138	105	97	124	85	89	g/l	110 – 160
Hematocrit	49,8	37,7	29,4	45,3	34,7	28,7	41,4	32,7	28,9	37,2	24,5	26,4	%	36 - 48
Platelets	213	216	333	284	241	550	249	259	373	184	200	373	10^9 /l	150 – 400
Band cells	0	0	3	0	2	1	1	4	3	1	4	1	%	1 – 5
Segmented neutrophils	3,49	10,4	5,86	3,48	12,53	5,09	4,11	11,91	4,49	3,03	9,36	7,11	10^9 /l	1,9 – 6,6
Eosinophils	0	4	8	0	2	1	1	3	1	1	1	1	%	1 - 5
Basophils	0	1	0	0	0	0	0	0	0	0	0	0	%	0 – 1
Lymphocytes	35,9	13,9	19,5	43,9	9,1	28,8	28,2	6,3	33,3	29,8	8,5	14,4	%	19 - 37
Monocytes	5,0	6,8	3,7	5,6	3,4	5,4	6,5	5,7	5,1	7,3	5,1	6,1	%	3 - 11
ESR	2	3	16	4	4	8	2	5	5	3	7	9	mm/h	1 -30

Table S2. Muscle damage biomarker levels of patients in the control and experimental rehabilitation groups 1 day before surgery (0), 1 day (1) and 12 days after THA.

Blood parameters	Patient 1, control group			Patient 2, experimental group			Patient 3, experimental group			Patient 4, experimental group			Units of measure ment	Reference ranges
	0 day	1 day	12 days	0 day	1 day	12 days	0 day	1 day	12 days	1 day	1 day	12 days		
AST	11,14	21,79	12,11	23,01	43,11	14,77	24,7	55,7	19,37	19,62	32,45	36,76	U/L	5,0 – 40,0
LDH	312	402	373	314	377	384	298	390	491	286	412	449	U/L	240 – 480
CPK	47,1	927,9	70,1	108,5	959,1	62,4	109	1262,6	71,6	121,5	694,8	78,1	U/L	24 – 170
Myoglobin	16,6	807	20,5	33,9	612,3	44,9	51,2	502,4	33,1	29,4	346,1	41,1	ng/ml	14,3 – 65,8

Blood parameters	Patient 5, control group			Patient 6, control group			Patient 7, experimental group			Patient 8, control group			Units of measure ment	Reference ranges
	0 day	1 day	12 days	0 day	1 day	12 days	0 day	1 day	12 days	0 day	1 day	12 days		
AST	25,17	45,79	18,46	18,46	32,37	19,66	14,62	35,24	10,55	18,38	42,2	16,57	U/L	5,0 – 40,0
LDH	284	463	463	322	341	438	186	186	162	263	397	366	U/L	240 – 480
CPK	69,4	1910,3	121,5	63,6	787,7	49	104,7	783,6	50,0	73,6	1467,3	37,6	U/L	24 – 170
Myoglobin	15,9	1700,5	28,9	22,5	213,4	17,2	31,6	470,0	26,3	28,1	593,1	17,8	ng/ml	14,3 – 65,8

Table S3. Preoperative scoring and test results of patients in the control and experimental rehabilitation groups (measurements taken before surgery).

Patient's Number, group	Lequesne scale, points	Harris scale, points	«Timed Up and Go» test, seconds	10-metre walk test, seconds
Patient 1, control group	23,5	29	31,51	46,64
Patient 2, experimental group	13	64	26,07	20,13
Patient 3, experimental group	14,5	53	41,38	28,03
Patient 4, experimental group	13	66	13,06	13,73
Patient 5, control group	13,5	61	26,64	25,38
Patient 6, control group	8,5	74	11,75	12,51
Patient 7, experimental group	1	104	8,59	11,56
Patient 8, control group	18	46	34,57	33,09

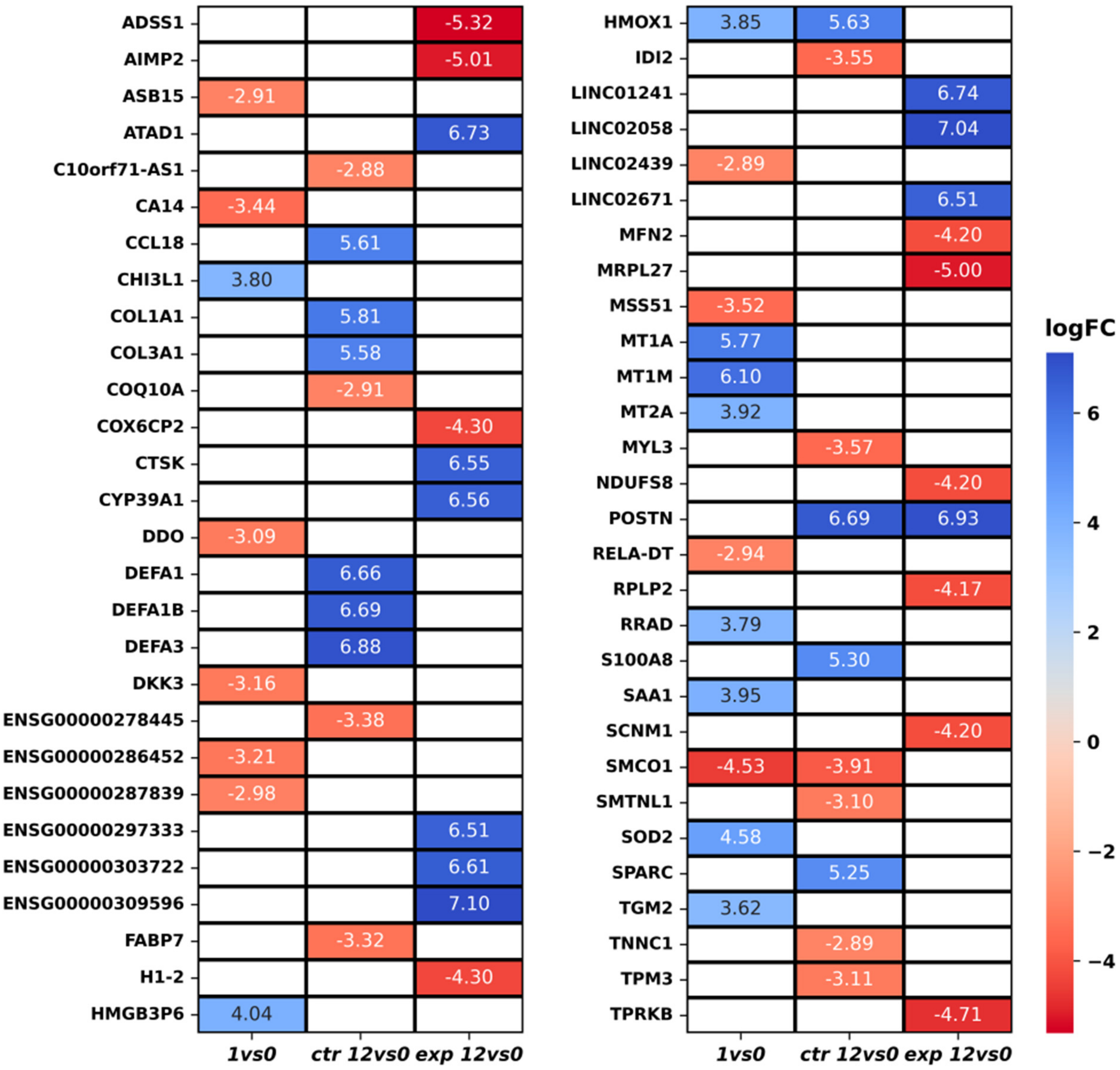

Figure S1. Genes with the Most Upregulated and Downregulated Expression in the 1 vs 0, ctr12 vs 0, and exp12 vs 0 Groups. FDR < 0.05.

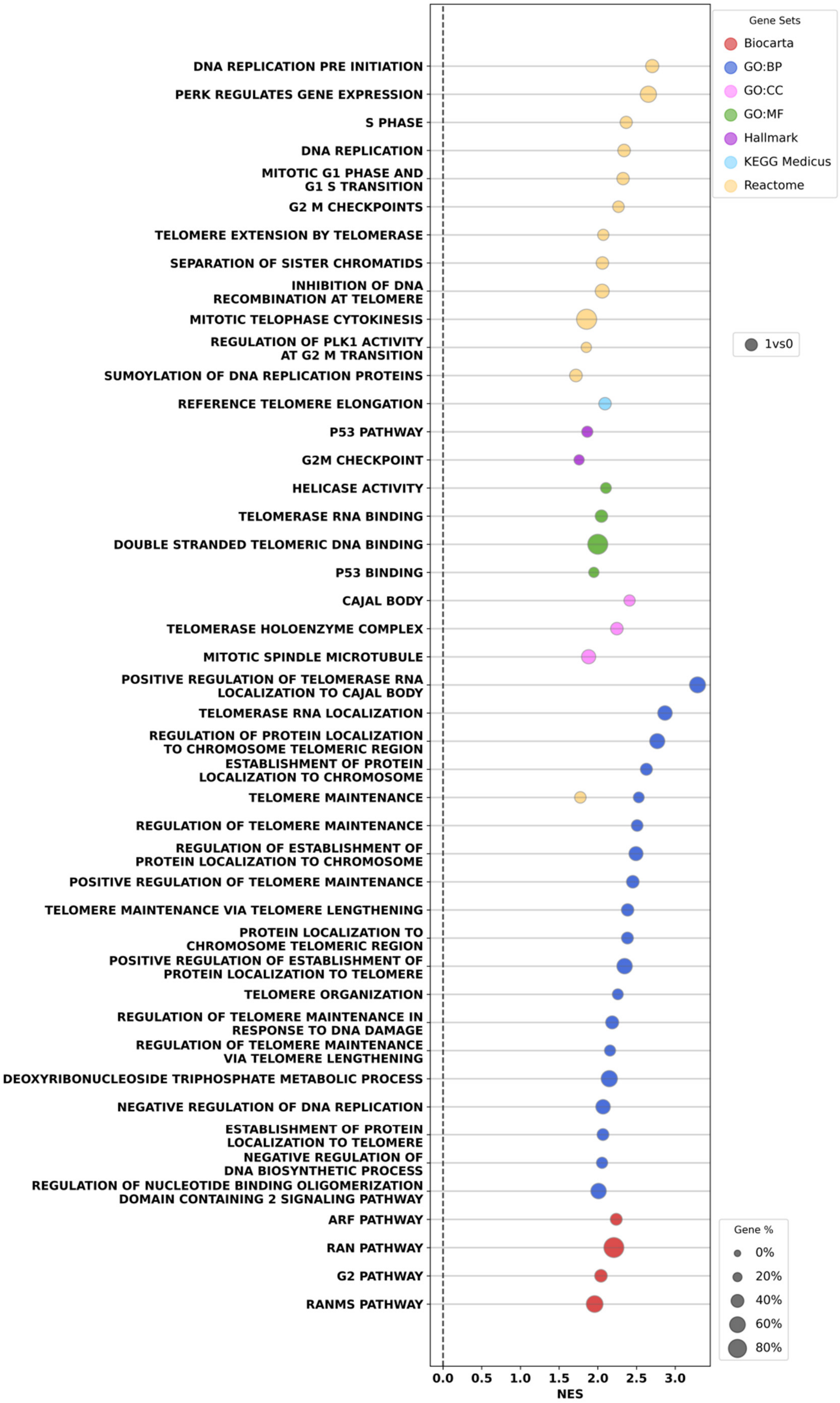
DNA REPLICATION PRE INITIATION
PERK REGULATES GENE EXPRESSION
S PHASE
DNA REPLICATION
MITOTIC G1 PHASE AND G1 S TRANSITION
G2 M CHECKPOINTS
TELOMERE EXTENSION BY TELOMERASE
SEPARATION OF SISTER CHROMATIDS
INHIBITION OF DNA RECOMBINATION AT TELOMERE
MITOTIC TELOPHASE CYTOKINESIS
REGULATION OF PLK1 ACTIVITY AT G2 M TRANSITION
SUMOYLATION OF DNA REPLICATION PROTEINS
REFERENCE TELOMERE ELONGATION
P53 PATHWAY
G2M CHECKPOINT
HELICASE ACTIVITY
TELOMERASE RNA BINDING
DOUBLE STRANDED TELOMERIC DNA BINDING
P53 BINDING
CAJAL BODY
TELOMERASE HOLOENZYME COMPLEX
MITOTIC SPINDLE MICROTUBULE
POSITIVE REGULATION OF TELOMERASE RNA LOCALIZATION TO CAJAL BODY
TELOMERASE RNA LOCALIZATION
REGULATION OF PROTEIN LOCALIZATION TO CHROMOSOME TELOMERIC REGION
ESTABLISHMENT OF PROTEIN LOCALIZATION TO CHROMOSOME
TELOMERE MAINTENANCE
REGULATION OF TELOMERE MAINTENANCE
REGULATION OF ESTABLISHMENT OF PROTEIN LOCALIZATION TO CHROMOSOME
POSITIVE REGULATION OF TELOMERE MAINTENANCE
TELOMERE MAINTENANCE VIA TELOMERE LENGTHENING
PROTEIN LOCALIZATION TO CHROMOSOME TELOMERIC REGION
POSITIVE REGULATION OF ESTABLISHMENT OF PROTEIN LOCALIZATION TO TELOMERE
TELOMERE ORGANIZATION
REGULATION OF TELOMERE MAINTENANCE IN RESPONSE TO DNA DAMAGE
REGULATION OF TELOMERE MAINTENANCE VIA TELOMERE LENGTHENING
DEOXYRIBONUCLEOSIDE TRIPHOSPHATE METABOLIC PROCESS
NEGATIVE REGULATION OF DNA REPLICATION
ESTABLISHMENT OF PROTEIN LOCALIZATION TO TELOMERE
NEGATIVE REGULATION OF DNA BIOSYNTHETIC PROCESS
REGULATION OF NUCLEOTIDE BINDING OLIGOMERIZATION DOMAIN CONTAINING 2 SIGNALING PATHWAY
ARF PATHWAY
RAN PATHWAY
G2 PATHWAY
RANMS PATHWAY
0.0
0.5
1.0
1.5
2.0
2.5
3.0
NES
Gene Sets
Biocarta
GO:BP
GO:CC
GO:MF
Hallmark
KEGG Medicus
Reactome
1vs0
Gene %
0%
20%
40%
60%
80%

(a)

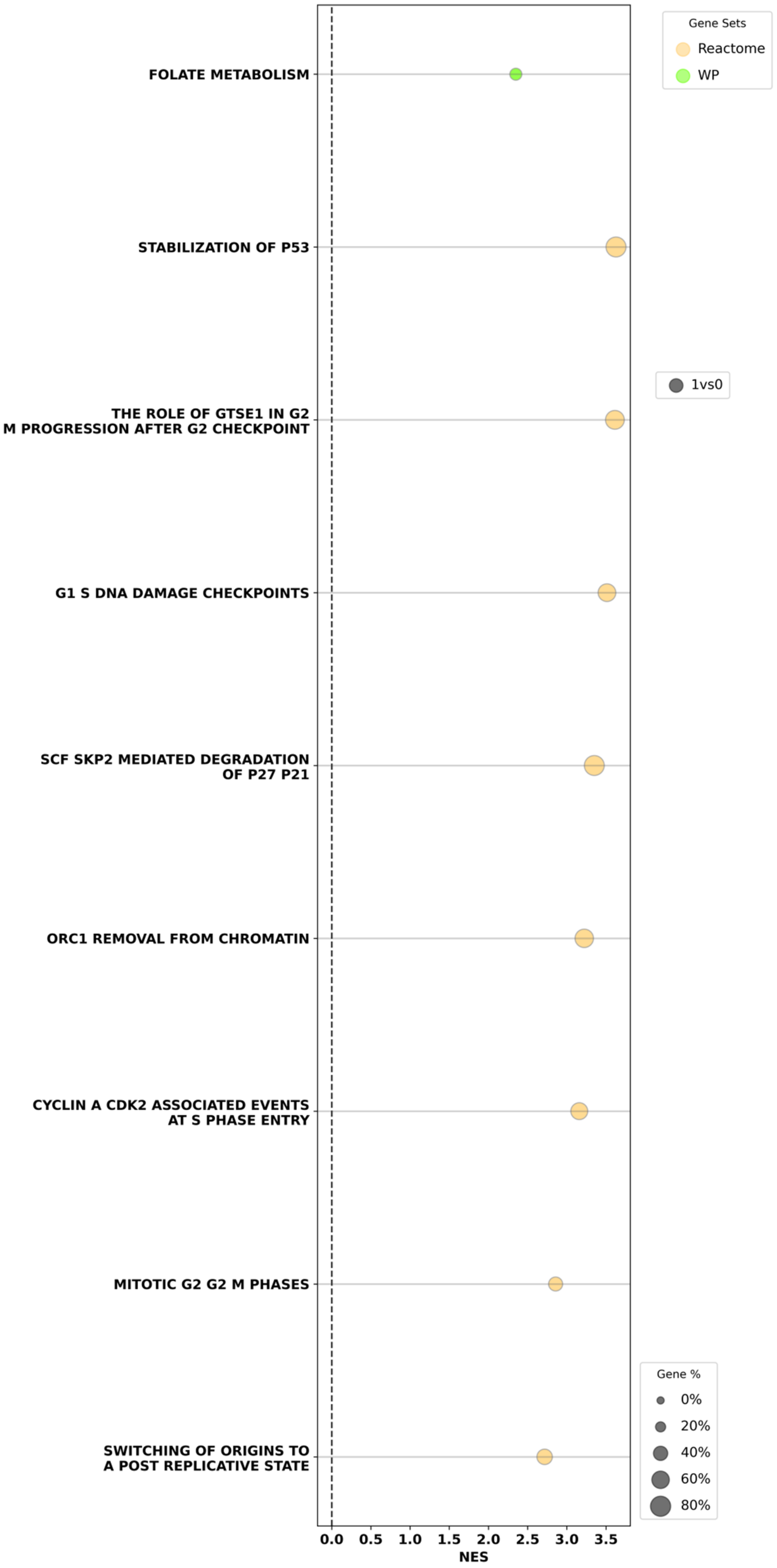

(b)

Figure S2. Gene Set Enrichment Analysis (GSEA) dot plots show activation of gene sets associated with the cell cycle (a), (b) in the rectus femoris 1 day after THA, compared to that at the start of the operation, in both control and experimental rehabilitation groups. NES – normalized enrichment score. Dot color denotes the different databases used in the analysis. Dot size reflects the percentage of identified genes per gene set.

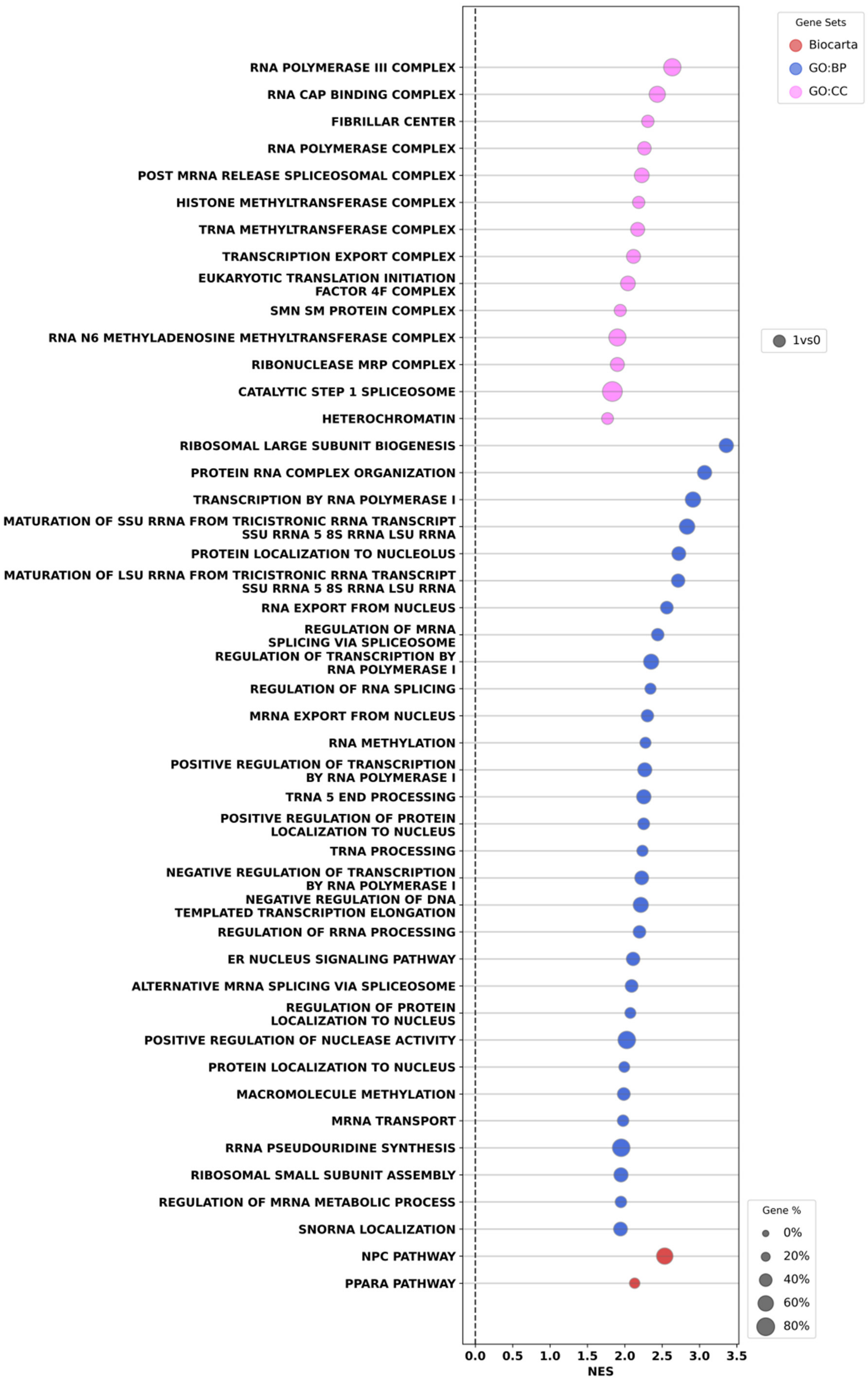

RNA POLYMERASE III COMPLEX
RNA CAP BINDING COMPLEX
FIBRILLAR CENTER
RNA POLYMERASE COMPLEX
POST MRNA RELEASE SPLICEOSOMAL COMPLEX
HISTONE METHYLTRANSFERASE COMPLEX
TRNA METHYLTRANSFERASE COMPLEX
TRANSCRIPTION EXPORT COMPLEX
EUKARYOTIC TRANSLATION INITIATION FACTOR 4F COMPLEX
SMN SM PROTEIN COMPLEX
RNA N6 METHYLADENOSINE METHYLTRANSFERASE COMPLEX
RIBONUCLEASE MRP COMPLEX
CATALYTIC STEP 1 SPLICEOSOME
HETEROCHROMATIN
RIBOSOMAL LARGE SUBUNIT BIOGENESIS
PROTEIN RNA COMPLEX ORGANIZATION
TRANSCRIPTION BY RNA POLYMERASE I
MATURATION OF SSU RRNA FROM TRICISTRONIC RRNA TRANSCRIPT SSU RRNA 5 8S RRNA LSU RRNA
PROTEIN LOCALIZATION TO NUCLEOLUS
MATURATION OF LSU RRNA FROM TRICISTRONIC RRNA TRANSCRIPT SSU RRNA 5 8S RRNA LSU RRNA
RNA EXPORT FROM NUCLEUS
REGULATION OF MRNA SPLICING VIA SPLICEOSOME
REGULATION OF TRANSCRIPTION BY RNA POLYMERASE I
REGULATION OF RNA SPLICING
MRNA EXPORT FROM NUCLEUS
RNA METHYLATION
POSITIVE REGULATION OF TRANSCRIPTION BY RNA POLYMERASE I
TRNA 5 END PROCESSING
POSITIVE REGULATION OF PROTEIN LOCALIZATION TO NUCLEUS
TRNA PROCESSING
NEGATIVE REGULATION OF TRANSCRIPTION BY RNA POLYMERASE I
NEGATIVE REGULATION OF DNA TEMPLATED TRANSCRIPTION ELONGATION
REGULATION OF RRNA PROCESSING
ER NUCLEUS SIGNALING PATHWAY
ALTERNATIVE MRNA SPLICING VIA SPLICEOSOME
REGULATION OF PROTEIN LOCALIZATION TO NUCLEUS
POSITIVE REGULATION OF NUCLEASE ACTIVITY
PROTEIN LOCALIZATION TO NUCLEUS
MACROMOLECULE METHYLATION
MRNA TRANSPORT
RRNA PSEUDOURIDINE SYNTHESIS
RIBOSOMAL SMALL SUBUNIT ASSEMBLY
REGULATION OF MRNA METABOLIC PROCESS
SNORNA LOCALIZATION
NPC PATHWAY
PPARA PATHWAY
0.0 0.5 1.0 1.5 2.0 2.5 3.0 3.5
NES
Gene Sets
Biocarta
GO:BP
GO:CC
1vs0
Gene %
0%
20%
40%
60%
80%

(a)

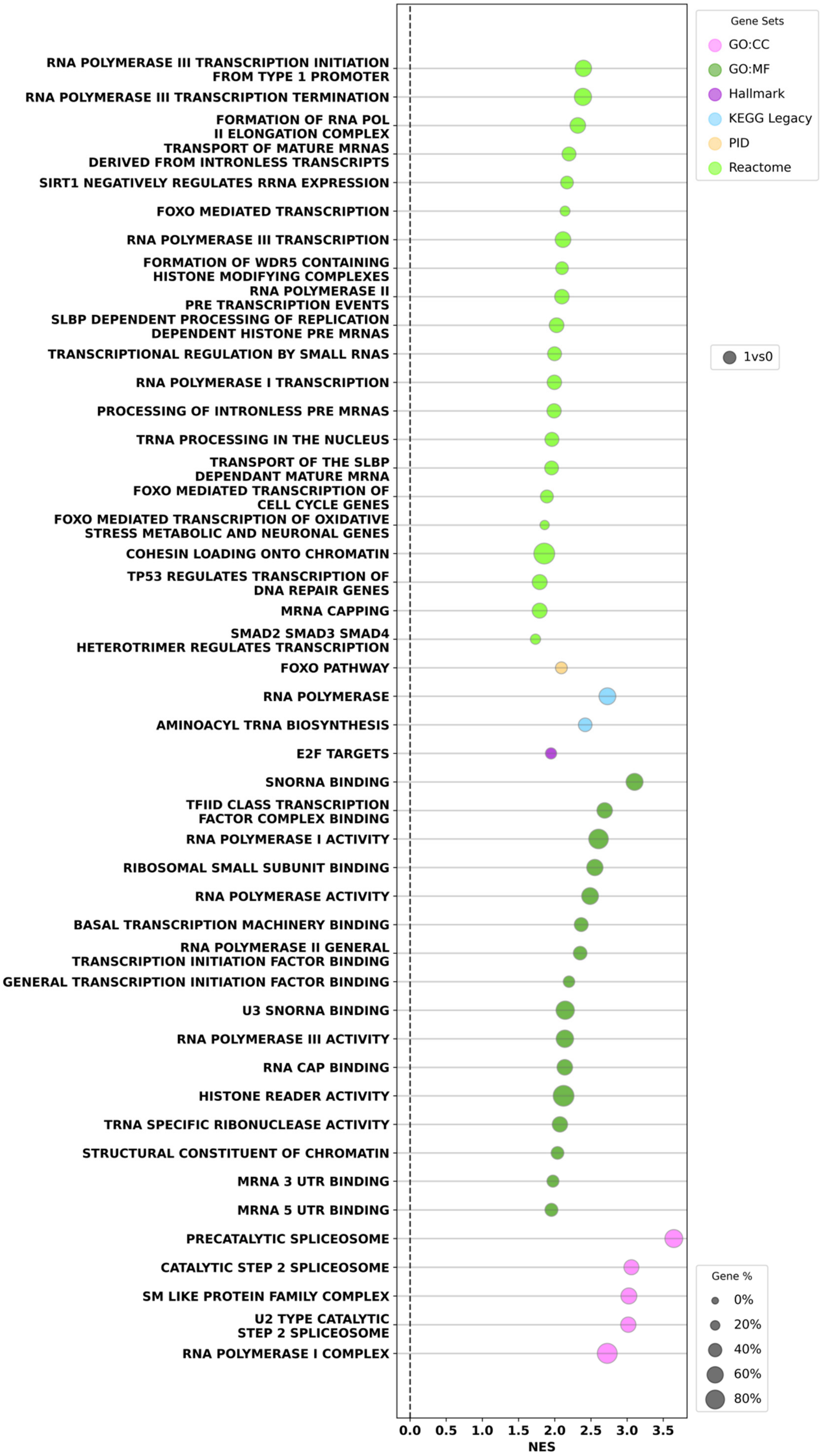

RNA POLYMERASE III TRANSCRIPTION INITIATION FROM TYPE 1 PROMOTER
RNA POLYMERASE III TRANSCRIPTION TERMINATION
FORMATION OF RNA POL II ELONGATION COMPLEX
TRANSPORT OF MATURE MRNAS DERIVED FROM INTRONLESS TRANSCRIPTS
SIRT1 NEGATIVELY REGULATES RRNA EXPRESSION
FOXO MEDIATED TRANSCRIPTION
RNA POLYMERASE III TRANSCRIPTION
FORMATION OF WDR5 CONTAINING HISTONE MODIFYING COMPLEXES
RNA POLYMERASE II PRE TRANSCRIPTION EVENTS
SLBP DEPENDENT PROCESSING OF REPLICATION DEPENDENT HISTONE PRE MRNAS
TRANSCRIPTIONAL REGULATION BY SMALL RNAS
RNA POLYMERASE I TRANSCRIPTION
PROCESSING OF INTRONLESS PRE MRNAS
TRNA PROCESSING IN THE NUCLEUS
TRANSPORT OF THE SLBP DEPENDANT MATURE MRNA
FOXO MEDIATED TRANSCRIPTION OF CELL CYCLE GENES
FOXO MEDIATED TRANSCRIPTION OF OXIDATIVE STRESS METABOLIC AND NEURONAL GENES
COHESIN LOADING ONTO CHROMATIN
TP53 REGULATES TRANSCRIPTION OF DNA REPAIR GENES
MRNA CAPPING
SMAD2 SMAD3 SMAD4 HETEROTRIMER REGULATES TRANSCRIPTION
FOXO PATHWAY
RNA POLYMERASE
AMINOACYL TRNA BIOSYNTHESIS
E2F TARGETS
SNORNA BINDING
TFIID CLASS TRANSCRIPTION FACTOR COMPLEX BINDING
RNA POLYMERASE I ACTIVITY
RIBOSOMAL SMALL SUBUNIT BINDING
RNA POLYMERASE ACTIVITY
BASAL TRANSCRIPTION MACHINERY BINDING
RNA POLYMERASE II GENERAL TRANSCRIPTION INITIATION FACTOR BINDING
GENERAL TRANSCRIPTION INITIATION FACTOR BINDING
U3 SNORNA BINDING
RNA POLYMERASE III ACTIVITY
RNA CAP BINDING
HISTONE READER ACTIVITY
TRNA SPECIFIC RIBONUCLEASE ACTIVITY
STRUCTURAL CONSTITUENT OF CHROMATIN
MRNA 3 UTR BINDING
MRNA 5 UTR BINDING
PRECATALYTIC SPLICEOSOME
CATALYTIC STEP 2 SPLICEOSOME
SM LIKE PROTEIN FAMILY COMPLEX
U2 TYPE CATALYTIC STEP 2 SPLICEOSOME
RNA POLYMERASE I COMPLEX
0.0 0.5 1.0 1.5 2.0 2.5 3.0 3.5
NES
Gene Sets
GO:CC
GO:MF
Hallmark
KEGG Legacy
PID
Reactome
1vs0
Gene %
0%
20%
40%
60%
80%

(b)

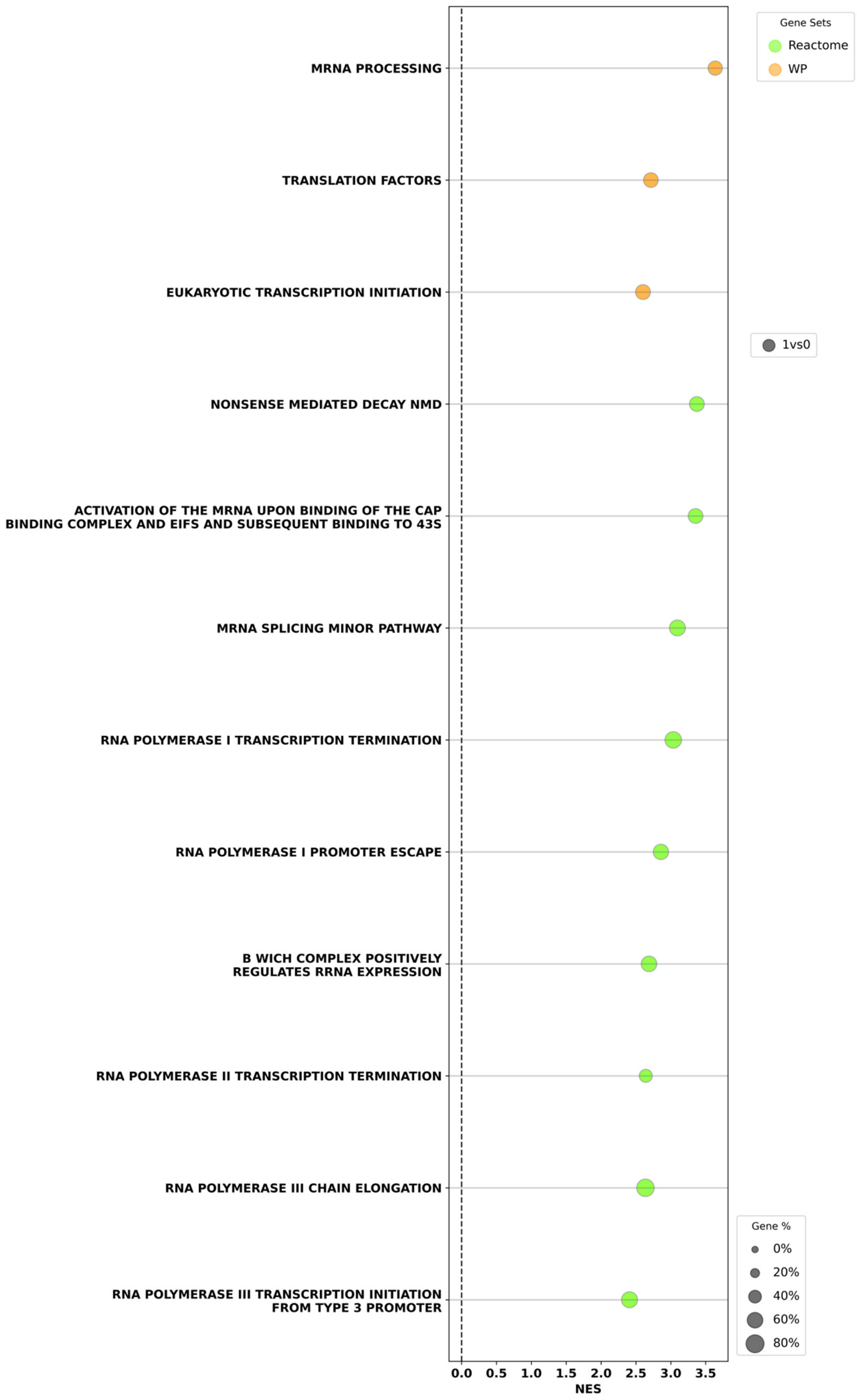

(c)

Figure S3. GSEA dot plots show activation of gene sets associated with the gene transcription (a), (b) and (c) in the rectus femoris 1 day after THA, compared to that at the start of the operation, in both control and experimental rehabilitation groups. NES – normalized enrichment score. Dot color denotes the different databases used in the analysis. Dot size reflects the percentage of identified genes per gene set.

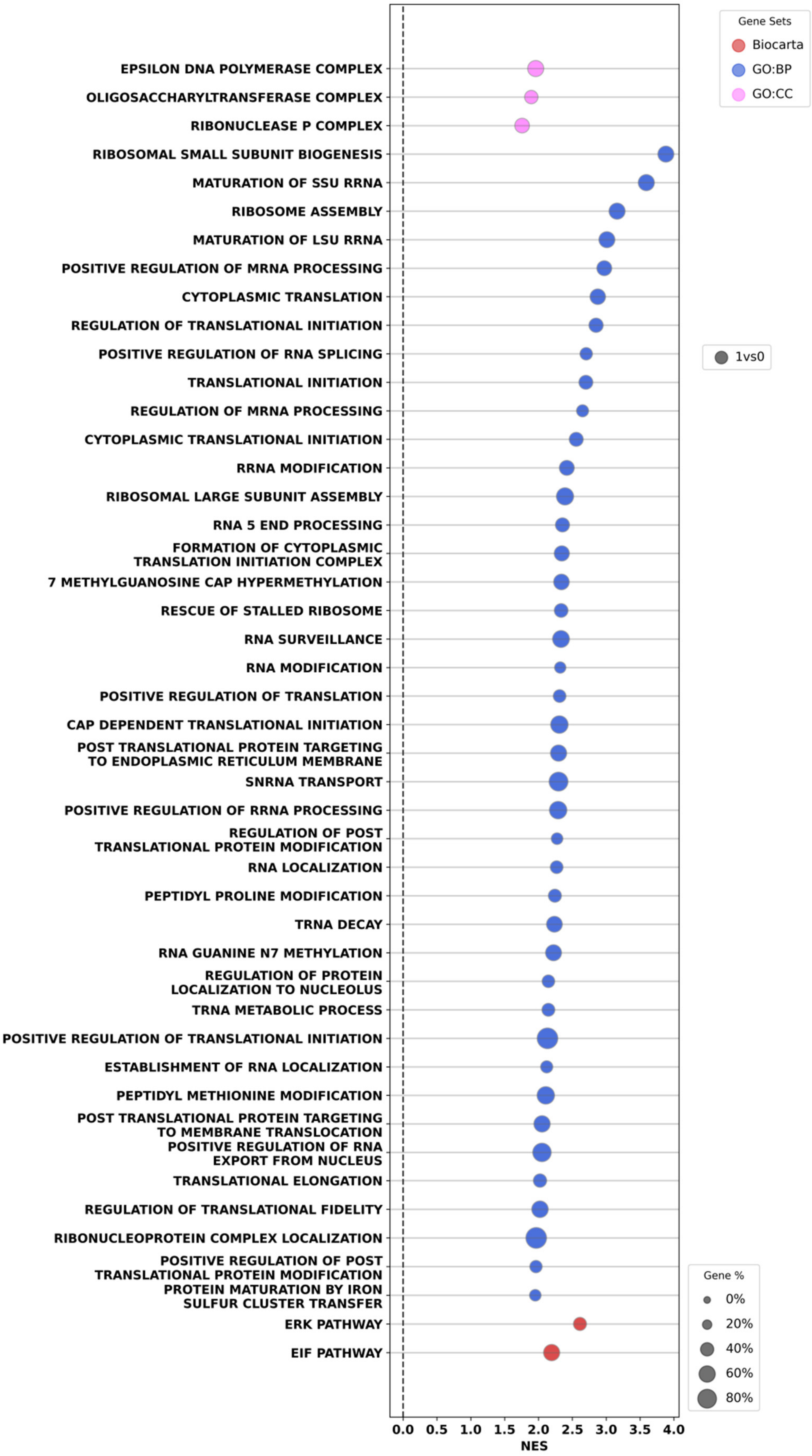
EPSILON DNA POLYMERASE COMPLEX
OLIGOSACCHARYLTRANSFERASE COMPLEX
RIBONUCLEASE P COMPLEX
RIBOSOMAL SMALL SUBUNIT BIOGENESIS
MATURATION OF SSU RRNA
RIBOSOME ASSEMBLY
MATURATION OF LSU RRNA
POSITIVE REGULATION OF MRNA PROCESSING
CYTOPLASMIC TRANSLATION
REGULATION OF TRANSLATIONAL INITIATION
POSITIVE REGULATION OF RNA SPLICING
TRANSLATIONAL INITIATION
REGULATION OF MRNA PROCESSING
CYTOPLASMIC TRANSLATIONAL INITIATION
RRNA MODIFICATION
RIBOSOMAL LARGE SUBUNIT ASSEMBLY
RNA 5 END PROCESSING
FORMATION OF CYTOPLASMIC TRANSLATION INITIATION COMPLEX
7 METHYLGUANOSINE CAP HYPERMETHYLATION
RESCUE OF STALLED RIBOSOME
RNA SURVEILLANCE
RNA MODIFICATION
POSITIVE REGULATION OF TRANSLATION
CAP DEPENDENT TRANSLATIONAL INITIATION
POST TRANSLATIONAL PROTEIN TARGETING TO ENDOPLASMIC RETICULUM MEMBRANE
SNRNA TRANSPORT
POSITIVE REGULATION OF RRNA PROCESSING
REGULATION OF POST TRANSLATIONAL PROTEIN MODIFICATION
RNA LOCALIZATION
PEPTIDYL PROLINE MODIFICATION
TRNA DECAY
RNA GUANINE N7 METHYLATION
REGULATION OF PROTEIN LOCALIZATION TO NUCLEOLUS
TRNA METABOLIC PROCESS
POSITIVE REGULATION OF TRANSLATIONAL INITIATION
ESTABLISHMENT OF RNA LOCALIZATION
PEPTIDYL METHIONINE MODIFICATION
POST TRANSLATIONAL PROTEIN TARGETING TO MEMBRANE TRANSLOCATION
POSITIVE REGULATION OF RNA EXPORT FROM NUCLEUS
TRANSLATIONAL ELONGATION
REGULATION OF TRANSLATIONAL FIDELITY
RIBONUCLEOPROTEIN COMPLEX LOCALIZATION
POSITIVE REGULATION OF POST TRANSLATIONAL PROTEIN MODIFICATION
PROTEIN MATURATION BY IRON SULFUR CLUSTER TRANSFER
ERK PATHWAY
EIF PATHWAY
0.0 0.5 1.0 1.5 2.0 2.5 3.0 3.5 4.0
NES
Gene Sets
Biocarta
GO:BP
GO:CC
1vs0
Gene %
0%
20%
40%
60%
80%

(a)

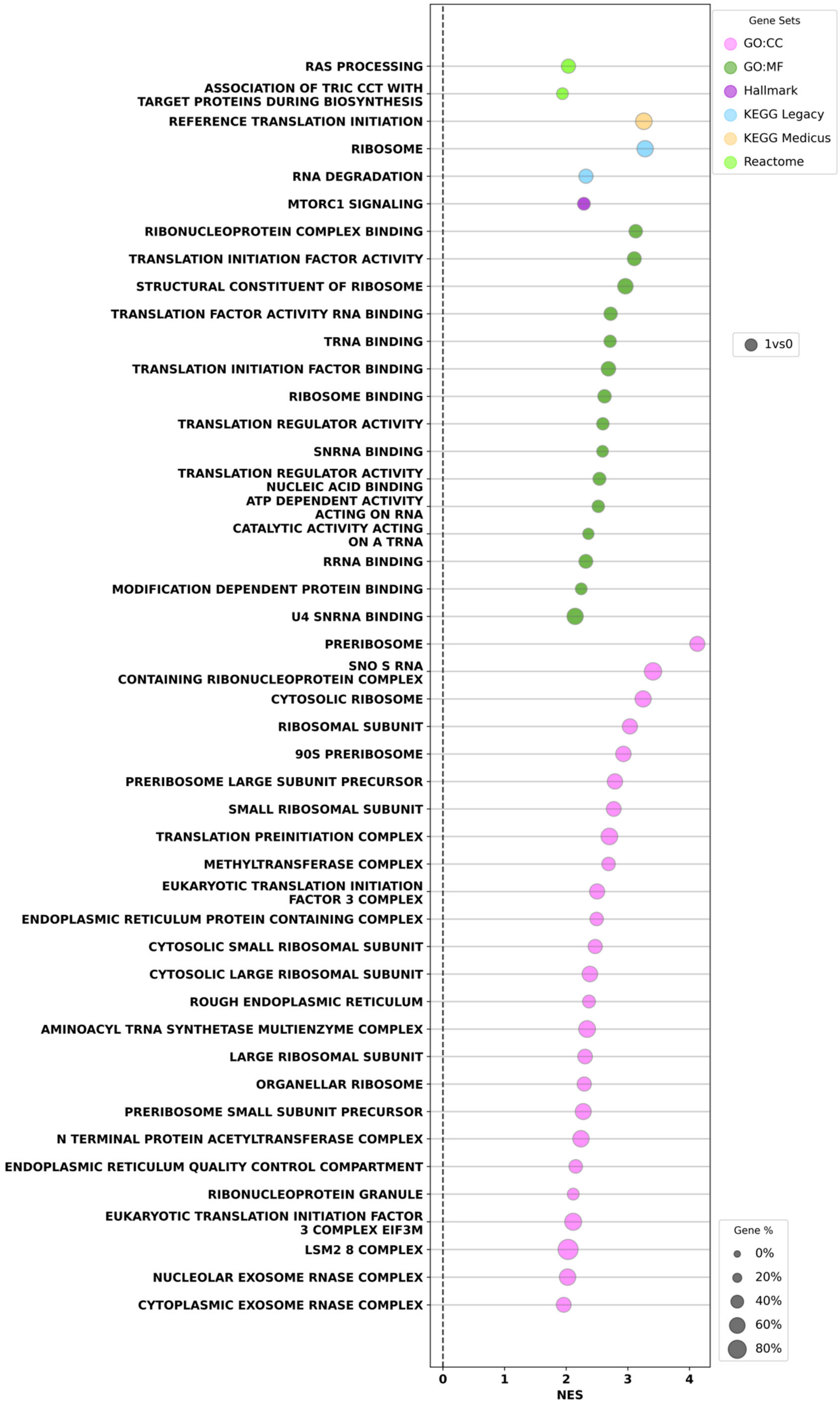
RAS PROCESSING
ASSOCIATION OF TRIC CCT WITH TARGET PROTEINS DURING BIOSYNTHESIS
REFERENCE TRANSLATION INITIATION
RIBOSOME
RNA DEGRADATION
MTORC1 SIGNALING
RIBONUCLEOPROTEIN COMPLEX BINDING
TRANSLATION INITIATION FACTOR ACTIVITY
STRUCTURAL CONSTITUENT OF RIBOSOME
TRANSLATION FACTOR ACTIVITY RNA BINDING
TRNA BINDING
TRANSLATION INITIATION FACTOR BINDING
RIBOSOME BINDING
TRANSLATION REGULATOR ACTIVITY
SNRNA BINDING
TRANSLATION REGULATOR ACTIVITY NUCLEIC ACID BINDING
ATP DEPENDENT ACTIVITY ACTING ON RNA
CATALYTIC ACTIVITY ACTING ON A TRNA
RRNA BINDING
MODIFICATION DEPENDENT PROTEIN BINDING
U4 SNRNA BINDING
PRERIBOSOME
SNO S RNA CONTAINING RIBONUCLEOPROTEIN COMPLEX
CYTOSOLIC RIBOSOME
RIBOSOMAL SUBUNIT
90S PRERIBOSOME
PRERIBOSOME LARGE SUBUNIT PRECURSOR
SMALL RIBOSOMAL SUBUNIT
TRANSLATION PREINITIATION COMPLEX
METHYLTRANSFERASE COMPLEX
EUKARYOTIC TRANSLATION INITIATION FACTOR 3 COMPLEX
ENDOPLASMIC RETICULUM PROTEIN CONTAINING COMPLEX
CYTOSOLIC SMALL RIBOSOMAL SUBUNIT
CYTOSOLIC LARGE RIBOSOMAL SUBUNIT
ROUGH ENDOPLASMIC RETICULUM
AMINOACYL TRNA SYNTHETASE MULTIENZYME COMPLEX
LARGE RIBOSOMAL SUBUNIT
ORGANELLAR RIBOSOME
PRERIBOSOME SMALL SUBUNIT PRECURSOR
N TERMINAL PROTEIN ACETYLTRANSFERASE COMPLEX
ENDOPLASMIC RETICULUM QUALITY CONTROL COMPARTMENT
RIBONUCLEOPROTEIN GRANULE
EUKARYOTIC TRANSLATION INITIATION FACTOR 3 COMPLEX EIF3M
LSM2 8 COMPLEX
NUCLEOLAR EXOSOME RNASE COMPLEX
CYTOPLASMIC EXOSOME RNASE COMPLEX
0
1
2
3
4
NES
Gene Sets
GO:CC
GO:MF
Hallmark
KEGG Legacy
KEGG Medicus
Reactome
1vs0
Gene %
0%
20%
40%
60%
80%

(b)

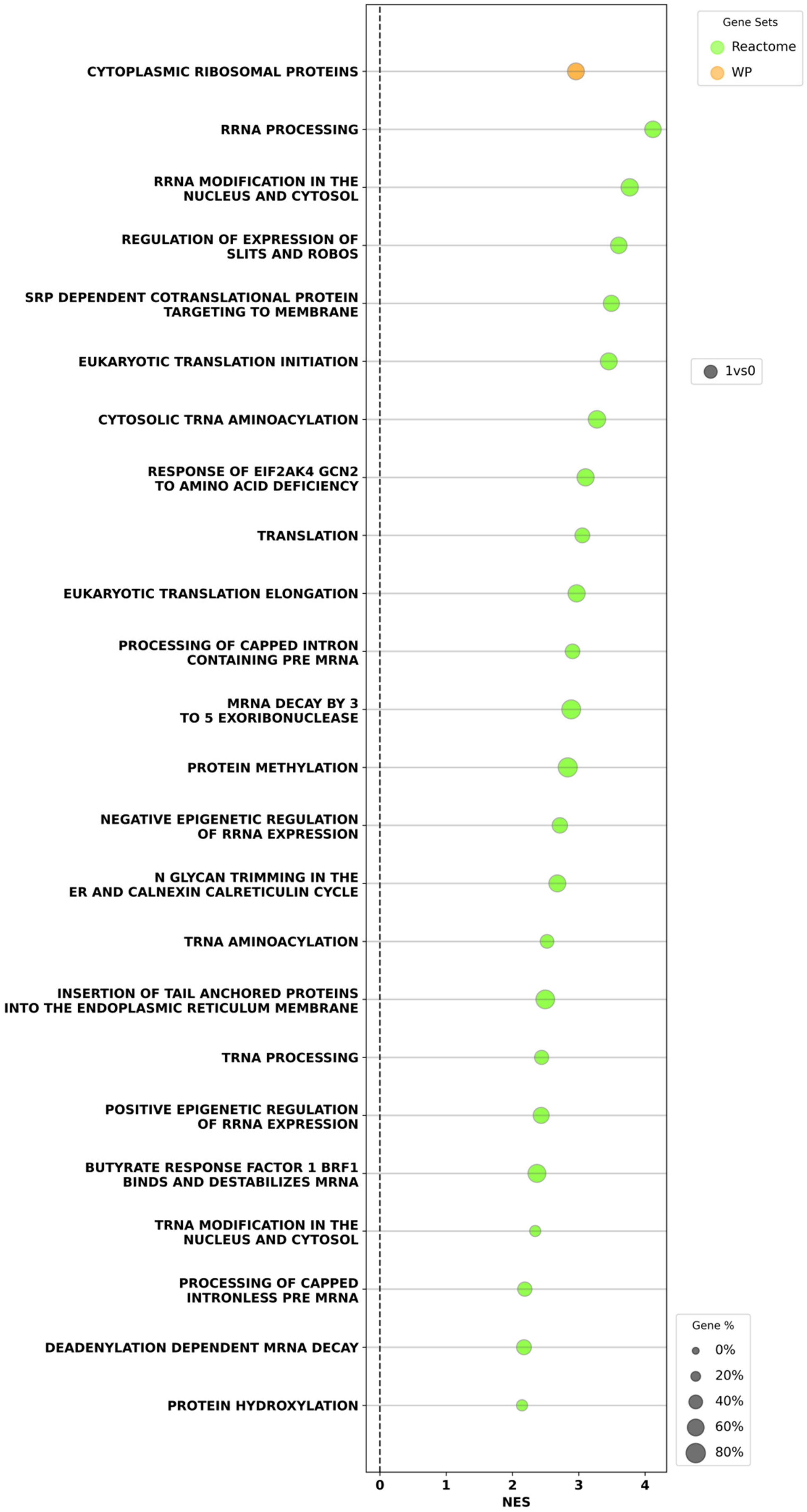

CYTOPLASMIC RIBOSOMAL PROTEINS
RRNA PROCESSING
RRNA MODIFICATION IN THE NUCLEUS AND CYTOSOL
REGULATION OF EXPRESSION OF SLITS AND ROBOS
SRP DEPENDENT COTRANSLATIONAL PROTEIN TARGETING TO MEMBRANE
EUKARYOTIC TRANSLATION INITIATION
CYTOSOLIC TRNA AMINOACYLATION
RESPONSE OF EIF2AK4 GCN2 TO AMINO ACID DEFICIENCY
TRANSLATION
EUKARYOTIC TRANSLATION ELONGATION
PROCESSING OF CAPPED INTRON CONTAINING PRE MRNA
MRNA DECAY BY 3 TO 5 EXORIBONUCLEASE
PROTEIN METHYLATION
NEGATIVE EPIGENETIC REGULATION OF RRNA EXPRESSION
N GLYCAN TRIMMING IN THE ER AND CALNEXIN CALRETICULIN CYCLE
TRNA AMINOACYLATION
INSERTION OF TAIL ANCHORED PROTEINS INTO THE ENDOPLASMIC RETICULUM MEMBRANE
TRNA PROCESSING
POSITIVE EPIGENETIC REGULATION OF RRNA EXPRESSION
BUTYRATE RESPONSE FACTOR 1 BRF1 BINDS AND DESTABILIZES MRNA
TRNA MODIFICATION IN THE NUCLEUS AND CYTOSOL
PROCESSING OF CAPPED INTRONLESS PRE MRNA
DEADENYLATION DEPENDENT MRNA DECAY
PROTEIN HYDROXYLATION
0
1
2
3
4
NES
Gene Sets
Reactome
WP
1vs0
Gene %
0%
20%
40%
60%
80%

(c)

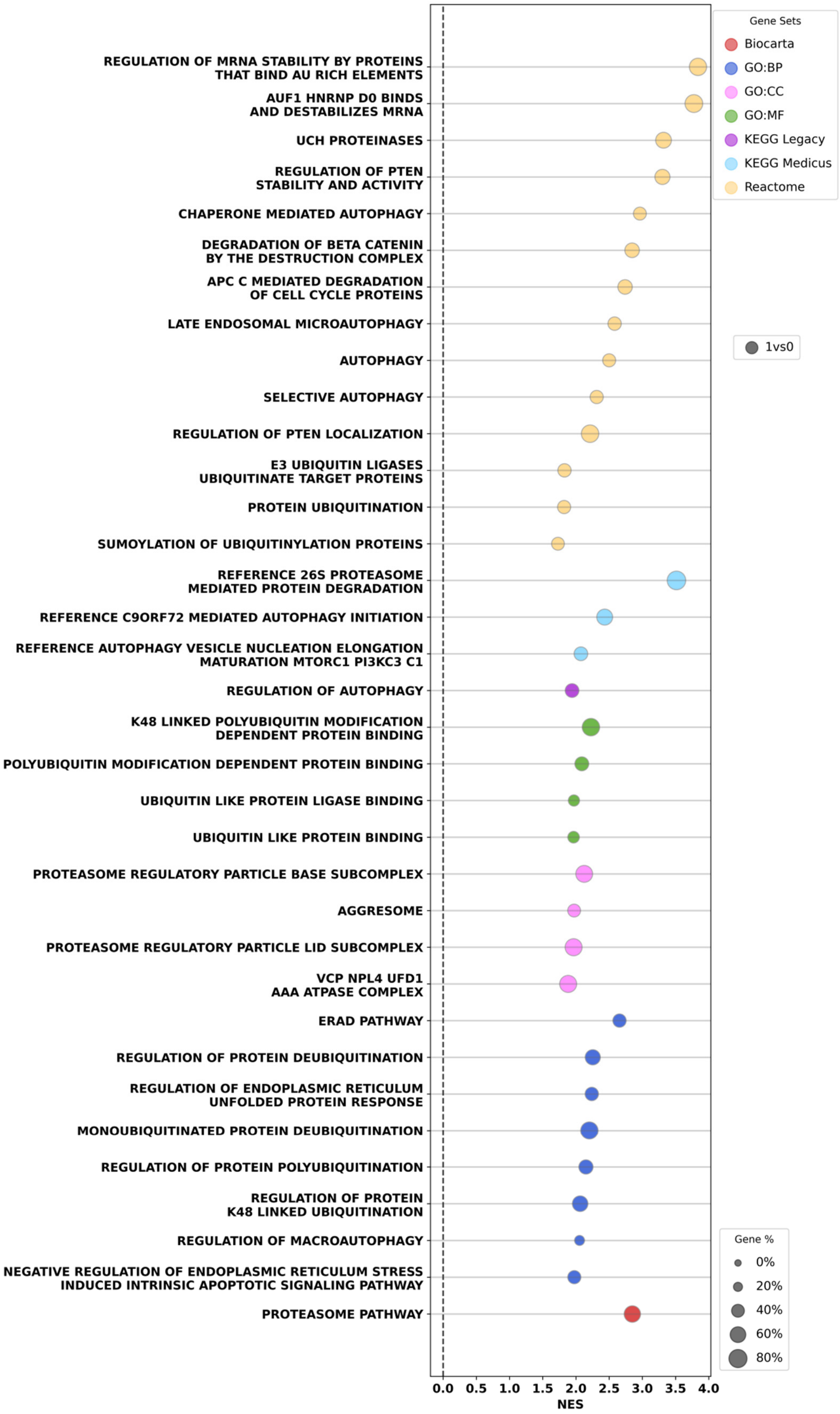

(d)

Figure S4. GSEA dot plots show activation of gene sets associated with the protein synthesis (a), (b), (c) and the protein degradation (d) in the rectus femoris 1 day after THA, compared to that at the start of the operation, in both control and experimental rehabilitation groups. NES – normalized enrichment score. Dot color denotes the different databases used in the analysis. Dot size reflects the percentage of identified genes per gene set.

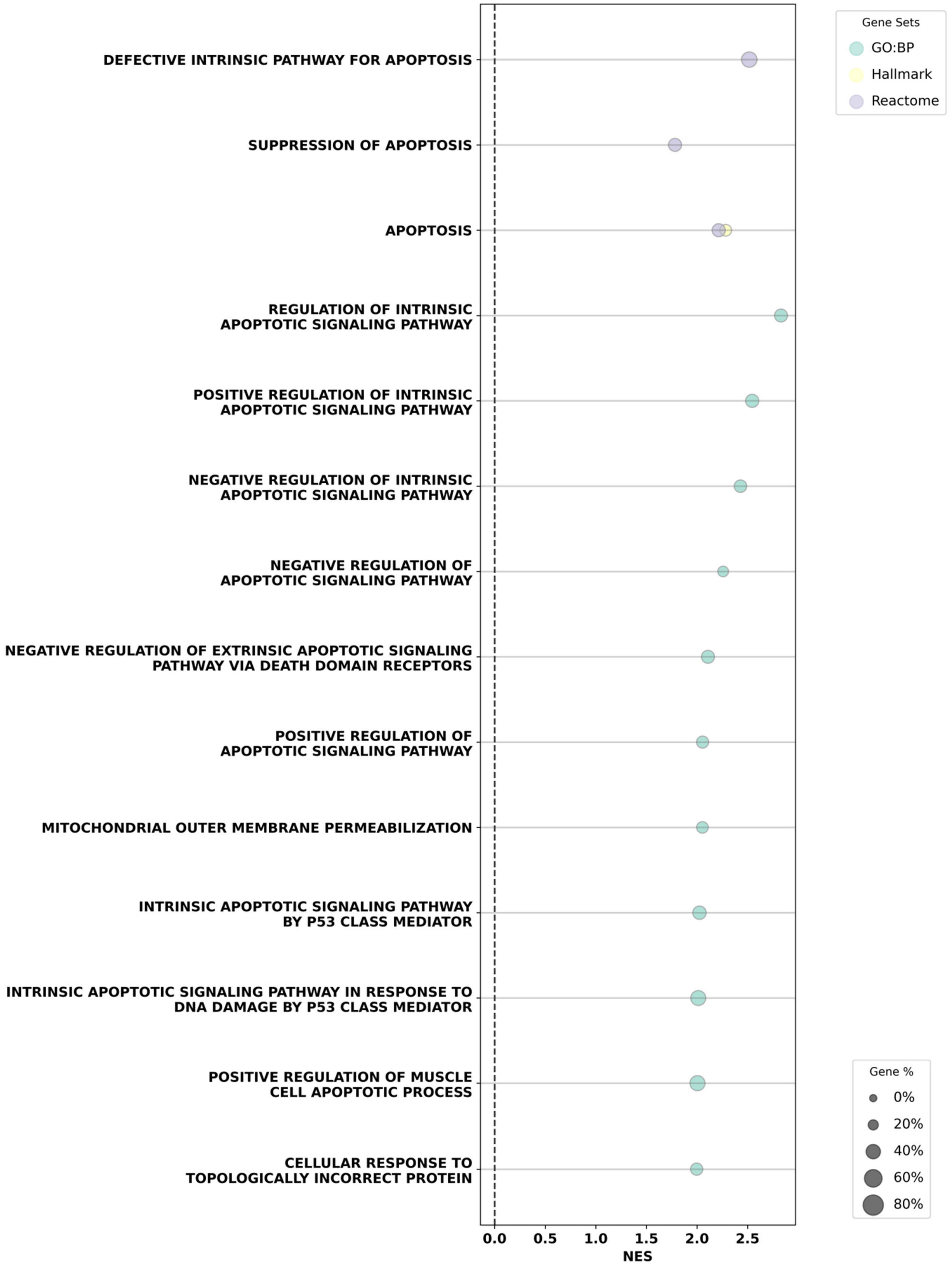

Figure S5. GSEA dot plots show activation of gene sets associated the apoptosis and autophagy in the rectus femoris 1 day after THA, compared to that at the start of the operation, in both control and experimental rehabilitation groups. NES – normalized enrichment score. Dot color denotes the different databases used in the analysis. Dot size reflects the percentage of identified genes per gene set.
