# Supplementary figures and images for: Molecular Indicators of Isometric Exercise Efficacy in Early Rehabilitation of Older Adults After Total Hip Arthroplasty
Source: Int J Mol Sci. 2026 Jan 30;27(3):1389. doi: 10.3390/ijms27031389 (PMC12898363; doi:10.3390/ijms27031389)

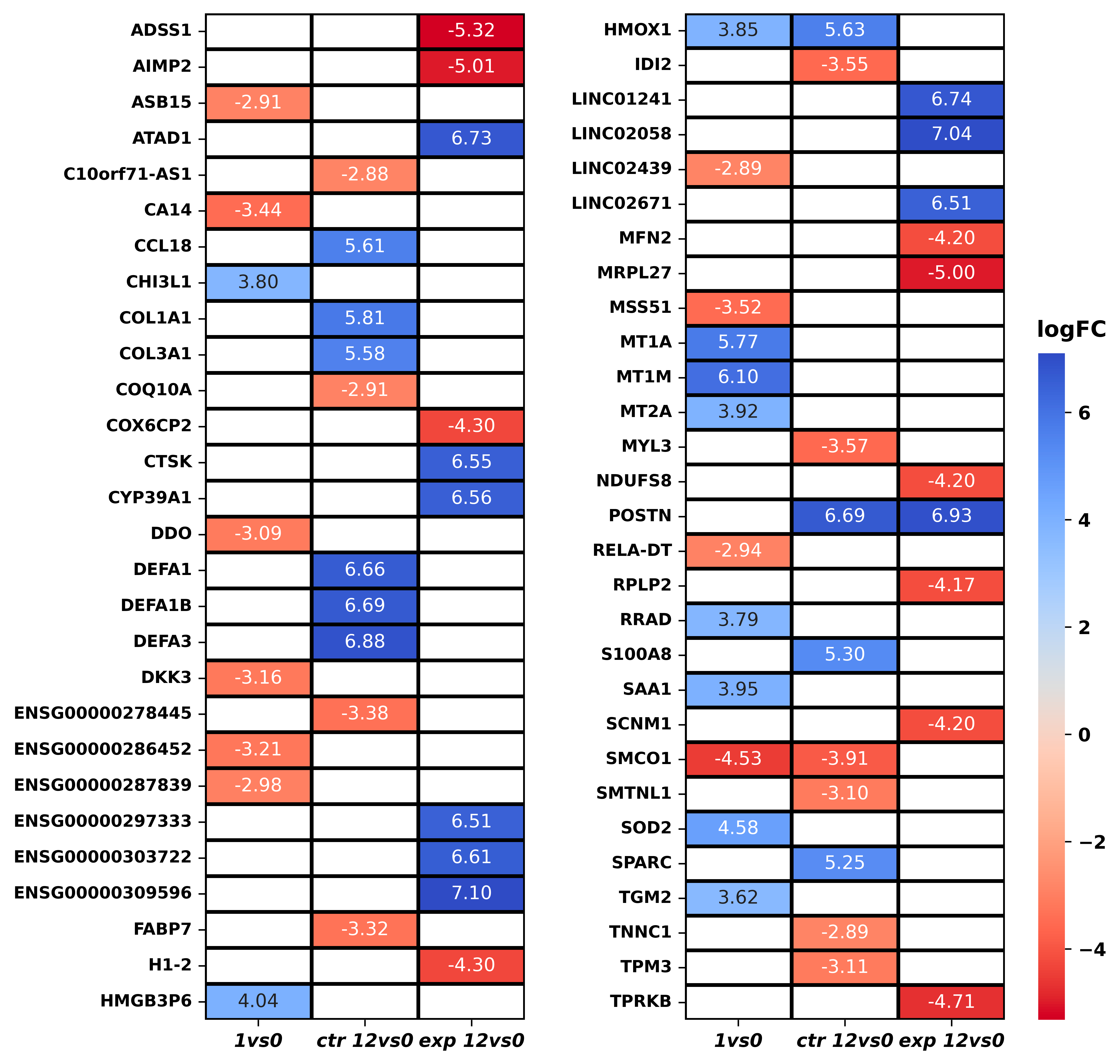

Supplement: Supplementary file 1 [file ijms-27-01389-s001.zip › Figure S1.png]
